# Outcome measurement tools in pediatric oncology palliative care: a scoping review of domains, validation and contextual relevance

**DOI:** 10.1038/s44276-026-00219-9

**Published:** 2026-06-16

**Authors:** Vani Verma, Arun Ghoshal, Mebin Mathew, Harshitha D, Aishwarya Sanian, Krithika S. Rao, Vasudeva Bhat K, Naveen Salins

**Affiliations:** 1 Department of Palliative Medicine and Supportive Care, Kasturba Medical College, Manipal, Manipal Academy of Higher Education, Manipal, India; 2 Department of Pediatric Oncology, Kasturba Medical College, Manipal, Manipal Academy of Higher Education, Manipal, India

## Abstract

Paediatric palliative care (PPC) in oncology plays a crucial role in enhancing the quality of life for children with cancer and their families. However, outcome measurement in this context remains fragmented, with tools often lacking validation, paediatric specificity, or contextual relevance, especially in low- and middle-income countries (LMICs). This scoping review maps the outcome measurement tools used in paediatric oncology palliative care, examining their assessed domains, psychometric properties and cultural, linguistic and health-system contextual relevance, particularly in LMICs. We systematically searched six databases from 2006 to 2025 and identified 27 eligible studies reporting on 28 unique tools across six key domains: symptom burden, quality of life, psychological well-being, spiritual health, caregiver burden and end-of-life care. While tools like Paediatric Quality of Life Inventory (PedsQL) and Symptom Screening in Pediatrics Tool (SSPedi) showed strong psychometric performance, only a few had been adapted for LMIC contexts. The psychological and spiritual domains were underrepresented, and most tools relied on proxy reporting, which limited child-centred assessments. The findings suggest the need for validated, culturally sensitive and inclusive tools co-developed with children and caregivers. Establishing such outcome measures is essential to ensuring equitable, high-quality palliative care across diverse settings. This review lays the groundwork for developing tools and advancing policies that support comprehensive and compassionate care for children with cancer. Review registration: The review is registered on Open Science Framework (OSF) 10.17605/OSF.IO/G8BN3.

## Introduction

Paediatric palliative care (PPC) provides holistic support for children with life-limiting illnesses and their families, aiming to alleviate physical, emotional, social and spiritual distress [1]. In oncology, PPC plays a pivotal role when introduced early, as it improves communication, reduces unnecessary interventions and enhances quality of life. Globally, an estimated 21 million children could benefit from PPC, with 8 million requiring specialised services [1]. Yet, measuring the impact of PPC remains complex, especially in oncology, where disease trajectories are unpredictable [2].

Children with cancer experience multifaceted symptom burdens, such as pain, nausea, fatigue and anxiety, which influence their functioning and emotional health [3, 4]. The emotional state of caregivers is closely linked to the child’s symptom experience [5]. Rigid coping strategies and feelings of helplessness among caregivers can lead to prolonged psychological distress [6].

In low-resource settings, structural barriers such as parental migration or delayed diagnosis exacerbate suffering. Despite increased awareness, palliative care is often introduced late, leaving many needs unmet [7]. Early identification of palliative care needs is essential [8]. Tools such as the Paediatric Distress Thermometer and the Paediatric Palliative Screening Scale (PaPaS) can support timely referrals [9, 10].

Patient-reported outcomes (PROs) are gaining prominence in paediatric oncology. Children expressing their symptoms in their own words, particularly through digital tools, enhances real-time care [11, 12]. Yet, these approaches remain underutilized, especially in advanced cancers like diffuse intrinsic pontine glioma, where symptom intensity is high [13].

Although several outcome measurement tools exist, many lack specificity for paediatric oncology [14]. Tools are often adapted from adult settings, with limited validation in children and rare contextualisation for low- and middle-income countries (LMICs) [14–18]. While culturally appropriate and cancer-specific tools can enhance accurate assessment and equitable care delivery, well-validated, generic tools (e.g. Patient Reported Outcomes Measurement Information System (PROMIS) paediatric measures) may also effectively capture key domains when supported by strong evidence for content validity, reliability, responsiveness and clinical actionability.

This scoping review maps the outcome measurement tools used in paediatric oncology palliative care. It focuses on domains assessed, psychometric rigor, applicability to children and caregivers and relevance to low-resource settings. Our goal is to inform tool development and promote equitable, comprehensive care.

## Materials and methods

This scoping review was conducted using the methodological framework proposed by Arksey and O’Malley, refined by Levac et al. later [19–22]. The following six steps: (1) identifying the research question; (2) identifying relevant studies; (3) study selection; (4) charting the data; (5) collating, summarising and reporting the results; (6) consultation exercise. The scoping review report followed the Preferred Reporting Items for Systematic Reviews extension for Scoping Reviews (PRISMA-ScR) checklist [23] (Please see Supplementary File I). Any deviations from the published and registered protocol (10.17605/OSF.IO/G8BN3) have been documented and described accordingly.

### Stage 1: Identifying the research question

The first stage of the Arksey and O’Malley framework involves clearly defining the research question to guide the scope and direction of the review. Given the exploratory nature of this study, the review was structured around two broad key questions:

1. What outcome measurement tools are used in paediatric oncology palliative care?
2. What are the psychometric properties and contextual applicability of these tools?

These questions were developed to systematically map the available evidence on tools used to assess outcomes in children with cancer receiving palliative care, and to evaluate the extent to which these tools are validated and applicable across diverse settings.

### Stage 2: Identifying relevant studies

To ensure a focused and relevant evidence base, eligibility criteria were framed using the Population-Concept-Context (PCC) model. We included peer-reviewed empirical studies (quantitative, qualitative, or mixed-methods) published in English between January 1, 2006, and January 31, 2025, that involved children (0–18 years) diagnosed with cancer and receiving palliative or supportive care, regardless of whether they were enroled in a formal PPC programme. January 1, 2006, was selected because modern PPC outcome literature expanded significantly following the publication of the IMPaCCT standards in 2007 [24]. Studies were eligible if they described the development, validation, or application of outcome measurement tools assessing domains such as symptom burden, quality of life, psychological distress, caregiver burden, communication and spiritual needs. The domains listed (e.g. symptom burden, quality of life, psychological distress) were illustrative examples; any study reporting an outcome measurement tool in PPC oncology was eligible for inclusion. We also included studies involving family members, caregivers, or healthcare professionals reporting proxy outcomes, recognising the critical role of family-centred and patient-centred care in paediatric oncology. Only peer-reviewed studies published in English were included. Eligible studies included outcome tools used in routine clinical care, randomised controlled trials, observational studies and studies on the development or validation of tools. Studies that included participants over 18 years were retained if paediatric-specific data could be clearly extracted.

Exclusions applied to grey literature, review articles (used only for reference screening), conference abstracts and studies unrelated to cancer or palliative care.

A comprehensive database search was developed in collaboration with an experienced information specialist. On March 6, 2025, six major databases, PubMed (MEDLINE), Scopus, Embase, CINAHL, Web of Science and PsycINFO, were searched using a combination of MeSH terms and keywords relevant to paediatric cancer, palliative care and outcome assessment. Boolean operators (‘AND’, ‘OR’) and database-specific syntax were applied to optimise retrieval. A full search strategy for PubMed is detailed in Supplementary File II.

### Stage 3: Study selection

After completing the final search across all selected databases, the citations were imported into Covidence for de-duplication, resulting in a cleaned set of records [25]. These were then managed within the platform for systematic and organised screening and review. To ensure our inclusion criteria were applied consistently, we began with a pilot screening where 10% of the citations were reviewed independently by three team members (VV, MM and HD). Any differences in judgement were discussed with senior investigators (NS and AG), helping us refine and clarify our screening approach.

Following this, all remaining titles and abstracts were screened independently by two reviewers using Covidence’s random assignment tool. Records that appeared eligible were then retrieved for full-text screening, which was again conducted independently and blindly by two reviewers. Any disagreements at this stage were resolved through team discussions with senior members (NS and AG), resulting in a final, consensus-based selection of studies for inclusion.

### Stage 4: Charting the data

A structured data extraction form was thoughtfully designed by the review team to systematically capture all essential details aligned with the study objectives. The form was customised to gather comprehensive information from each included study, including author details, year of publication, country, study design, participant characteristics, cancer type, palliative care setting, the name and type of outcome measurement tool used, the domains it assessed (such as symptoms, quality of life, or caregiver burden), and any psychometric properties reported (like reliability and validity). The extraction process was carried out using Covidence’s Extraction 2.0 platform. For each study, data were extracted by one reviewer and independently verified by a second, who either confirmed their accuracy or made necessary corrections [25]. Once all studies were processed, the data were exported into Microsoft Excel for further analysis. Senior reviewers (NS and AG) conducted a final review of the dataset to ensure that all included studies adhered to the eligibility criteria and that each outcome measurement tool was correctly categorised in line with the review’s scope.

### Stage 5: Collating, summarising and reporting results

In line with our scoping review protocol, we initially planned to use the first two stages of thematic synthesis to analyze the findings. However, given the diversity and volume of data encountered, we adopted a more practical approach, organising the extracted information using predefined categories from our data extraction framework. This strategy enabled the creation of a structured and meaningful summary of outcome measurement tools used in paediatric oncology palliative care. Each study was analysed individually, even if multiple publications stemmed from the same dataset.

The tools were grouped based on the primary outcome domains they assessed, including symptom burden, quality of life, psychological well-being, caregiver burden and communication, and were presented in summary tables for clarity. Our extraction of psychometric properties was guided by COSMIN checklist (COnsensus-based Standards for the selection of health status Measurement INstruments), including reliability, validity, responsiveness, and cross-cultural adaptation [26].

### Stage 6: Consultation

Although formal stakeholder consultation was not undertaken, the findings were reviewed by PPC experts for their contextual and clinical relevance.

## Results

### Study characteristics

**Fig. 1:**
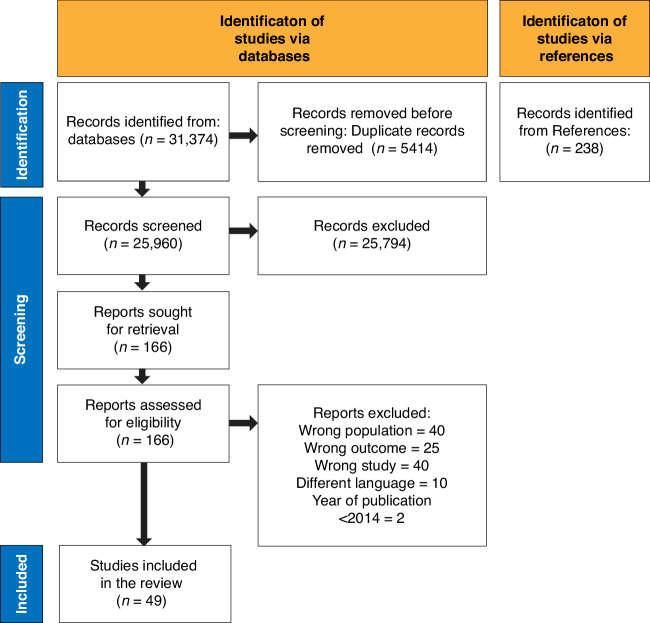
PRISMA 2020 flow diagram of study selection process. The diagram summarizes the process of study identification, screening, eligibility assessment, and inclusion based on predefined criteria. Records were identified from database searches and reference screening, followed by duplicate removal and sequential screening stages. Reasons for exclusion at the full-text stage are indicated within the figure.

A total of 31,374 records were identified from six databases. On removing 5414 duplicates, 25960 records were screened. Of these, 166 full-text articles were assessed for eligibility and 49 studies met the inclusion criteria (Fig. 1).

Table 1 provides an overview of the studies included. A total of 49 studies were included in this scoping review, which were published between 2007 and 2025.

**Table 1:** Overview of included studies (*n* = 49).

S.no	Authors & year	Country	Study title	Age range	Setting	Study design	Summary of findings	Practical implications	Study limitations
1	Murti Andriastuti et al. 2024	Indonesia	Family functioning, parental cancer-related emotions and quality of life in childhood cancer patients	2–18 years old	Hospital	Likely cross-sectional/observational	Family functioning & parental stress affect QoL	Address family dynamics & parental emotions	Sample size, reliance on parent reporting
2	Banayat, Campos, et al. 2024	Philippines	Care Needs of Parents of Children with Cancer	Parents of children with cancer	Hospital	Embedded mixed-method design	Practical needs (financial, housing) were top priorities. Emotional and spiritual needs are also high. Access to support groups and financial aid is critical.	Addressing financial, social support and spiritual needs enhances holistic, individualised care for the whole family during the cancer treatment journey.	The sample size for the qualitative phase is relatively small (*n* = 16), which limits the depth of qualitative data collected. Most participants were from low-income backgrounds.
3	Cowfer et al. 2024	USA	Relationships Between Parental Anxiety and Child Quality of Life in Advanced Childhood Cancer	5–17 years	Hospital	Cross-sectional study	Higher parental anxiety correlates with lower child psychosocial HRQoL, particularly in emotional and school functioning.	Regular assessments of parental mental health should be integrated into care for families dealing with advanced paediatric cancer.	Small sample size limits generalisability; focus on a single institution; limited diversity in parental roles (mostly mothers).
4	Cadamuro et al. 2024	Brazil	Association between multiple symptoms and quality of life of paediatric patients with cancer in Brazil	2–18 years	Hospital	Descriptive, cross-sectional study	Symptoms significantly impacted QoL across all domains, particularly in the emotional domain.	Regular screening for symptoms and supportive interventions for emotional well-being are necessary.	Exclusion of patients with neuropsychiatric disorders and visual impairment may limit generalisability.
5	Grinde et al. 2024	USA	Symptom Adverse Events and Quality of Life of Children With Advanced Cancer: Results From a Longitudinal Study	2–18 years	Hospital	Longitudinal, prospective, descriptive	CNS tumour patients experienced poorer QoL. Symptom frequency of nausea, anxiety and fatigue had significant negative correlations with QoL.	The use of Ped PRO-CTCAE® and PedsQL™ in clinical practice and research can help evaluate the relationship between symptoms and QoL in children with cancer	Limited to English and Spanish literate participants; small sample size, validation needed for Spanish Ped PRO-CTCAE® version
6	Hamner et al. 2024	USA	Quality of Life Among Paediatric Patients With Cancer: Contributions of Time Since Diagnosis and Parental Chronic Stress	5–18 years	Hospital	Prospective, observational	Parental chronic stress contributes to reduced emotional, physical and social QoL beyond time since diagnosis (TSD).	Oncological treatment should include family-focused interventions to reduce parental stress for better QoL outcomes in paediatric patients	Sample size was too small to examine cancer types separately; study used a dichotomous variable (ALL vs. others), limiting cancer-type specificity
7	Wolfe et al. 2024	USA	Symptoms and Distress in Children With Advanced Cancer: Prospective Patient-Reported Outcomes from the PediQUEST Study	2–18 years	Hospital	Prospective cohort study (embedded in PediQUEST RCT)	Children with advanced cancer frequently report high levels of symptom distress; pain, fatigue and irritability are common, especially near end of life	Implementing intensive symptom management strategies is essential, especially for patients undergoing high-intensity therapy or experiencing disease progression	Small sample size; patients were followed up until death or end of data collection, limiting longer-term insights on symptom management dynamics
8	M Sarah et al. 2024	UK	Experiences of healthcare, including palliative care, of children with life-limiting and life-threatening conditions and their families: a longitudinal qualitative investigation	5–18 years	Community-based/multi-setting qualitative	Longitudinal qualitative interview study	Urgent need for child and family-centred healthcare, integrated palliative care	Importance of trusted relationships and better coordination in care	Small sample size, focused on one geographical region (West Midlands, UK)
9	Nina Mogensen et al. 2024	Sweden, Finland, Denmark	Quality of life in children and adolescents after treatment for acute lymphoblastic leukaemia	1–18 years	Hospital	Retrospective cohort study	Self-reported HRQoL was comparable to that of the reference population for older children. However, parent-reported toxicity during treatment was associated with significantly lower total and physical HRQoL scores for younger children. Parent-proxy reports for younger children were lower across various domains. School functioning scores were particularly lower in high-risk patients, suggesting specific impacts of intensive treatments.	Minimising complications during treatment is crucial for improving long-term HRQoL. Targeted interventions for high-risk groups, especially concerning school functioning, are recommended.	Discrepancies in translation and cultural differences across countries (Denmark, Finland) may have impacted the school and social functioning scores. Parent-reported data may introduce bias. Removal of specific items from questionnaires limits comparability across countries.
10	Nair et al. 2024	India	Optimising Palliative Care for Children with Metastatic Neuroblastoma and the Paediatrician’s Role in a Shared Care Model	1–14 years	Hospital	Retrospective study based on case records	87 patients received palliative care consultation; early referral occurred in only 13 (14.9%) patients, pain was the primary symptom managed by the PC team. Nausea, constipation and abdominal distension were frequent at end-of-life	A shared care model can help ensure early PC referral, manage symptom burden, and provide psychosocial support throughout the cancer journey	Retrospective data, limited to one cancer centre, no specific QoL measurement tool, focus mainly on symptom burden and pain management
11	Pangarso et al. 2024	Indonesia	Discovering Needs for Palliative Care in Children with Cancer in Indonesia	Median age as a cutoff for younger vs. older children	Hospital	Cross-sectional study using semi-structured interviews	High prevalence of unmet physical, emotional and social needs; limited psychological support and insufficient communication with healthcare providers	Regular assessment of palliative care needs, better symptom management and active family involvement are crucial to improving care	Focused on one hospital; potential recall bias from parental reports; lack of quantitative QoL measurement
12	Brian T. Cheng, Tenzin Wangmo 2024	USA	Palliative care utilisation in hospitalised children with cancer	<18 years	Hospital	Cross-sectional	PC use was associated with shorter hospital stays, lower costs and varied by socioeconomic factors.	Highlights need for equitable PC access and better PC utilisation in paediatric oncology	Limited sensitivity of diagnostic codes for PC; retrospective analysis; focus on hospital-based data
13	Kimberley Widger et al. 2024	Canada	Predictors of Specialised Paediatric Palliative Care Involvement and Impact on Patterns of End-of-Life Care in Children With Cancer	0–18 years	Hospital	Retrospective cohort	SPPC involvement reduced high-intensity EOL care, but disparities were seen based on income, distance and cancer type.	Strong support for SPPC implementation to improve EOL outcomes and reduce disparities.	Retrospective design; limited to hospitals with SPPC teams; excluded patients without access to SPPC or within 30 days of diagnosis.
14	Sharp et al. 2023	USA	Predictors of psychological functioning in children with cancer: disposition and cumulative life stressors	8–17 years	Hospital	Cross-sectional study	Children’s psychological functioning is largely predicted by dispositional factors, with health status accounting for minimal variance. Demographic factors influenced anxiety and PTSS, with lower SES predicting higher symptoms.	Highlights importance of focusing on psychological disposition and life stressors in paediatric oncology.	Possible selection bias; differences in SES between groups; limited to English-speaking participants.
15	Zhong et al. 2023	China, Belgium	Chinese and Belgian paediatricians’ perspectives toward paediatric palliative care	Not applicable	Healthcare provider-focused perception study (online)	Cross-sectional survey	Chinese paediatricians face more personal and systemic obstacles; experience improves perception and delivery of PPC	Expanding PPC education and experience improves care delivery	Survey limitations (convenience sampling, self-reporting bias), cultural context and data primarily focused on paediatricians rather than direct patient-family needs.
16	Silva, A.V.M.V. et al. 2023	Brazil	Impact of childhood cancer on health-related quality of life in children and adolescents: self-perception and mother’s perception	1–18 years	Hospital	Cross-sectional study	Childhood cancer significantly impacts HRQoL, with notable differences between self-reported HRQoL of children/adolescents and their mothers’ perceptions.	Implement public health strategies focused on addressing HRQoL based on children’s self-reports	Small sample size and non-probabilistic sampling may limit generalisability. Potential biases in self-reporting and mothers’ perceptions may exist
17	Yang Chen et al. 2022	China	Palliative sedation for children at end of life: a retrospective cohort study	2–18 years	Hospital	Retrospective cohort study	Palliative sedation improves symptom control without impacting hospitalisation duration	Indicates the need for proper sedation protocols in paediatric palliative care	Single-centre study, limited generalisability
18	Trupti Narnaware, Maninderdeep Kaur, Sukhpal Kaur, Renu Madan 2022	India	Assessment of Quality of Life and Long-term Health-related Problems Among Children with Intracranial Tumour	4–18 years	Hospital	Descriptive study	Children experienced long-term health-related issues, including fatigue, neurocognitive dysfunction and endocrine problems. School functioning and physical health were the most affected domains	Interventions are necessary to enhance school and physical functioning after treatment. Long-term follow-up care is essential to mitigate health-related issues	The study was conducted in a single hospital, with self-reported data and a limited follow-up period. No home-based assessments or electronic tools used for data collection
19	Joana Duran, DrNP, et al. 2021	USA	Quality of Life and Pain Experienced by Children and Adolescents with Cancer at Home Following Discharge	8–17 years	Home based	Longitudinal, descriptive, exploratory.	Pain and fatigue negatively affect QoL, particularly physical and emotional functioning. Gender, age and cancer type influence outcomes.	Emphasises the need for tailored discharge plans addressing pain, fatigue and related QoL concerns for paediatric cancer patients transitioning home.	Small sample size, limited generalisability to non-English-speaking patients and lack of focus on spiritual and non-physical domains.
20	Hendriks et al. 2021	Switzerland	The unmet needs of childhood cancer survivors in long-term follow-up care	≥18 years	Hospital	Qualitative study with semistructured interviews	Childhood cancer survivors expressed unmet psychosocial needs, demand for centralised care and the importance of personalised long-term follow-up care.	Highlighted the importance of integrating psychosocial support into long-term follow-up care and providing personalised care services for survivors.	Small sample size, limited to Swiss residents, potential response bias from volunteers in the study.
21	Ghoshal A et al. 2021	India	Specialist Paediatric Palliative Care Referral Practices in Paediatric Oncology: A Large 5-year Retrospective Audit	≤18 years	Hospital	Retrospective 5-year audit.	Late referrals led to high symptom burden in patients. 21.2% continued oral metronomic chemotherapy, 10.5% were referred back to oncology for palliative radiotherapy and 90.8% were cared for at home.	Early referral to palliative care could help manage symptoms better and improve family support. Liaison with local healthcare providers is crucial for at-home end-of-life care.	The retrospective nature and missing data in some records may have impacted completeness of the findings. Limited to a single centre.
22	Erika Juškauskienė, RN et al. 2021	Lithuania	Spiritual Well-Being and Related Factors in Children With Cancer	5–12 years	Hospital	Cross-sectional quantitative study design.	Spiritual well-being is critical for the overall well-being of paediatric oncology patients; strong link between happiness and spiritual health observed.	Highlights need for incorporating spiritual care into paediatric oncology to enhance quality of life; understanding children’s perspectives is vital.	Limited generalisability due to the specific age range and cultural context; potential biases in self-reporting from children.
23	Erica C. Kaye et al. 2021	USA	Illness and End-of-Life Experiences of Children with Cancer Who Receive Palliative Care	2–18 years	Hospital	Retrospective cohort study.	Children receiving palliative care often face burdensome treatments and tend to die in intensive care settings; early palliative care involvement could improve their experiences.	Emphasises the importance of integrating palliative care early in treatment plans to enhance quality of life and potentially reduce distress at the end of life.	Limited to one academic center, which may affect generalisability; retrospective design may introduce bias in data collection and interpretation.
24	Mitchell S, 2021	UK	Experiences of healthcare, including palliative care, of children with life-limiting and life-threatening conditions and their families: a longitudinal qualitative investigation	5–18 years	Hospital	Longitudinal qualitative study	Participants described a fragmented healthcare experience with limited access to integrated palliative care, emphasising the importance of trusted relationships and the need for a more holistic approach to care.	Emphasises the importance of fostering interpersonal relationships and improving care coordination in paediatric palliative care settings.	Limited to specific geographical area, findings may not be generalisable and two children died during the study affecting the dynamics of the data.
25	Schneider A, Wurz A, Vogel A, 2021	Germany	Illness perceptions, fear of progression, and health-related quality of life during acute treatment and follow-up care in paediatric cancer patients and their parents: a cross-sectional study	4–18 years	Hospital	Cross-sectional study	Psychological factors play a crucial role in patients’ well-being, particularly in follow-up care, indicating a shift in the importance of sociodemographic and medical variables from acute treatment to long-term care.	Recommend standardised interventions to address maladaptive illness perceptions and include family-focused approaches.	Small sample size, non-random selection and potential bias in self-reported measures.
26	Russell et al. 2021	Canada	Psychosocial risk, symptom burden, and concerns in families affected by childhood cancer	8–18 years	Hospital	Cross-sectional descriptive, psychosocial assessment	Higher psychosocial risk at diagnosis predicted greater symptom burden and concerns during treatment; parents reported heightened concerns regarding practical and emotional needs	Ongoing psychosocial screening (using tools like PATrev, CPC) can help identify families needing targeted support early and throughout treatment	Limited to a single site in Canada, cross-sectional data cannot assess longitudinal changes in psychosocial risk, reliance on parent-reported data, not longitudinal
27	Song, I.G. et al. 2021	Korea	Paediatric palliative screening scale as a useful tool for clinicians’ assessment of palliative care needs of paediatric patients	1–18 years	Hospital	Retrospective cohort study	The PaPaS was effective in assessing palliative care needs, showing good agreement between clinicians and demonstrated reliability for use in paediatric care	Encouragement for primary care clinicians to utilise the PaPaS tool to enhance palliative care referrals and assessments.	Limitations include the study’s retrospective nature and the need for further validation of the PaPaS in varied clinical settings.
28	Robson, P. C., Dietrich, M. S., Akard, T. F. 2021	USA	Associations of Age, Gender and Family Income with Quality of Life in Children With Advanced Cancer	7–17 years	Hospital	Secondary analysis of RCT	The study highlights the impact of demographic characteristics on the quality of life of children with advanced cancer, emphasising the need for targeted interventions.	Identifying at-risk families can lead to interventions that improve QOL for children with advanced cancer.	Limited to cross-sectional evaluation at enrolment; no follow-up data.
29	Cheryl Rodgers et al. 2021	USA	Childhood Cancer Symptom Cluster: Leukaemia and Health-Related Quality of Life	3–18 years	Hospital	Prospective, repeated-measures	HRQOL scores improve over time; however, higher symptom severity in CCSC-L is associated with lower HRQOL, particularly from post-induction to maintenance therapy.	Nurses play a crucial role in implementing symptom management strategies that can alleviate symptoms and potentially improve HRQOL in children undergoing leukaemia treatment.	Limited to participants fluent in English or Spanish; excludes patients with cognitive disabilities established before diagnosis; variable follow-up due to treatment delays.
30	Jennifer L. Raybin et al. 2021	USA	Associations between demographics and quality of life in the first year of cancer treatment	3–17 years	Hospital	Cross-sectional secondary analysis of prospective data	No significant associations found with sex, race, SES, or distance. Increased worry, poor posture and decreased QoL are linked with age.	Potential use of posture and Faces Scale as non-invasive, accessible measures for QoL assessment in paediatric oncology.	Limited to English-speaking patients receiving chemotherapy. Biological markers are limited to posture alone.
31	Snaman j et al. 2020	Boston	Paediatric Palliative Care in Oncology	Not specified	Hospital	Review or conceptual framework likely; specific design not detailed.	The integration of palliative care into paediatric oncology can significantly enhance QoL; a multidisciplinary team is necessary to address the complex needs of patients and families.	Emphasises the need for early referral and a comprehensive approach to palliative care, focusing on the patient’s and family’s needs throughout the treatment trajectory.	The lack of quantitative data may limit the generalisability of findings; potential for selection bias in qualitative studies.
32	Nogueira A. et al. 2020	Portugal	The needs of children receiving end of life care and the impact of a paediatric palliative care team	1–18 years,	Hospital	Retrospective cohort study design to analyze existing clinical records.	Overview of the main findings, such as the prevalence of physical and emotional needs, the role of palliative care in improving quality of life and the resource use during the last year of life.	Recommendations for practice, such as the need for enhanced palliative care services, interdisciplinary approaches and training for healthcare providers in addressing the holistic needs of children with CCC.	Limitations of the study, such as potential biases in retrospective data, the focus on a single institution and the lack of a control group for comparisons.
33	Veronica Dussel et al. 2020	USA	Feasibility of Conducting a Palliative Care Randomised Controlled Trial in Children With Advanced Cancer: Assessment of the PediQUEST Study	2 years and older	Hospital	Cohort study embedded in an RCT	Conducting a PPC-RCT was feasible; however, 24% of participants experienced attrition due to death. Additionally, 41% of participants experienced intermittent attrition (IA) within the first 20 weeks, with a significant increase in IA during the last 8 weeks of life. Incentives, altruism, low-burden tasks and supportive staff were found to influence retention. Shortening follow-up to 3 months with re-enrolment improved recruitment by 20%	Multicenter PPC-RCT feasible if barriers like attrition at end-of-life are addressed; leveraging multicenter networks to broaden sample and adjusting follow-up may enhance study success	Limited to a few sites, resulting in a low average enrolment rate; single-country study reduces generalisability; retrospective analysis may not capture nuanced changes during PPC trial progression
34	Sung L, Zaoutis T, Ullrich CK, 2019	USA	Quality of Life in Paediatric Acute Myeloid Leukaemia: Report from the Children’s Oncology Group	2–18 years	Hospital	Prospective, multicenter study as part of AAML1031 trial	Decreased QoL was observed during chemotherapy, with significant impacts on pain, fatigue, anxiety and cognitive issues	Nurses and clinicians can focus on targeted QoL interventions during intensive chemotherapy phases	Study limited by missing data due to incomplete questionnaire responses, drop-out during the study and no electronic data collection method
35	Marieke Van Schoors et al. 2019	Belgium	Family Members Dealing With Childhood Cancer: A Study on the Role of Family Functioning and Cancer Appraisal	0–18 years	Community-based/Home-based (Survey-based longitudinal)	Longitudinal survey study	Family functioning and cancer appraisal significantly affect individual adjustment and QoL	Emphasises the need for interventions targeting both individual family members and the entire family unit	Limited to Caucasian, Dutch-speaking families, with possible cultural bias
36	Hamner, Latzman, Elkin, & Majumdar 2018	USA	Quality of Life Among Paediatric Patients With Cancer: Contributions of Time Since Diagnosis and Parental Chronic Stress	6–18 years (Mage = 10.2 ± 3.6)	Hospital	Observational study	Parental chronic stress impacts QoL across physical, emotional and social domains, independent of time since diagnosis	Highlights need for family-centred care and support for parental stress in paediatric oncology	Limited to single-center sample; results may not generalise to all demographic and socioeconomic backgrounds
37	Abby R. Rosenberg et al. 2016	USA	Quality of Life in Children With Advanced Cancer: A Report From the PediQUEST Study	2–18 years old	Hospital	Prospective cohort study	Quality of life deteriorates as cancer progresses; emotional distress and physical pain are common	Integrating palliative care earlier in the disease trajectory can address both physical and emotional needs, potentially improving QoL	Limited to children with advanced cancer; small sample size may limit generalisability; reliance on parent proxy reporting may skew QoL findings
38	Tamires Vieira Carneiro, 2016	Brazil	Quality of Life of Paediatric Oncology Patients	not specified	Hospital	Study design not specified; likely a cross-sectional study or qualitative assessment.	Key takeaways on the QoL of paediatric oncology patients and implications for palliative care services.	Suggestions for improving palliative care practices to enhance QoL outcomes for paediatric oncology patients.	Limitations not specified; could include sample size constraints, study design limitations, or reliance on parent reporting.
39	Momani T.G., Mandrell B.N., Gattuso J.S., et al. 2016	USA	Children’s Perspective on Health-Related Quality of Life during Active Treatment for Acute Lymphoblastic Leukaemia: An Advanced Content Analysis Approach	8–18 years	Hospital	Quantitative content analysis of qualitative data	Children’s HRQoL is influenced by cancer treatment, with responses varying by age, gender and risk group. Symptoms, social interactions, and emotional well-being are central themes. Adolescents tend to express more social needs, and females report more negative experiences. Some adolescents found positive personal growth through the illness experience.	Nurses can use these two questions to effectively assess the HRQoL of children and adolescents during ALL treatment and tailor interventions accordingly.	Limited to ALL and specific age group (8–18 years). Coding analysis software limitations, and small high-risk group. Data were collected over a long period (2002–2012), which may affect relevance to current treatments.
40	Olagunju et al. 2016	Nigeria	Child’s symptom burden and depressive symptoms among caregivers of children with cancers: an argument for early integration of paediatric palliative care	7–12 years	Hospital	Cross-sectional study	Caregivers experience significant depressive symptoms related to their children’s symptom burden, highlighting the need for better awareness and palliative care integration	Improve awareness to reduce late presentations and integrate palliative care for better symptom management and caregiver support.	Small sample size; limited generalisability; reliance on self-reported data.
41	Lubis et al. 2015	Indonesia	Quality of life in children with cancer and their normal siblings	5–18 years	Hospital	Cross-sectional study	Cancer significantly reduced QoL in all measured domains, with school function being the most impacted. No significant difference was found between haematologic malignancies and solid tumours.	Health professionals should provide additional support in areas such as education and emotional well-being for children undergoing cancer treatment.	Exclusion of children with mental retardation, remission, or severe malnutrition may have affected the representation of cancer types and patient conditions.
42	Veronica Dussel, MD, MPH, et al. 2015	USA	Feasibility of Conducting a Palliative Care Randomised Controlled Trial in Children With Advanced Cancer: Assessment of the PediQUEST Study	2 years and older	Hospital	Cohort study embedded in a pilot RCT.	PPC-RCTs are feasible, with post-inclusion retention proving adequate. Most families participated for altruistic reasons, and emotional support and trust in healthcare played roles. Shortening follow-up increased recruitment.	Large multicenter studies with sicker patients and strategies to improve data collection near the end of life could increase the feasibility of future PPC-RCTs.	Small sample size, potential attrition issues as disease progressed, challenges with late-stage patient participation, lack of focus on non-physical needs and intermittent attrition (IA).
43	Wolfe, J. 2015	USA	Symptoms and Distress in Children With Advanced Cancer: Prospective Patient-Reported Outcomes From the PediQUEST Study	2 years to 18	Hospital	Prospective cohort study embedded in a multisite randomised controlled trial	High prevalence of distressing symptoms in children with advanced cancer, intensified in later stages	Intensive symptom management strategies should be prioritised, especially during disease progression	Limited generalisability to other settings, potential biases in proxy reporting for younger children
44	Compas et al. 2014	USA	Children and Adolescents Coping With Cancer: Self- and Parent Reports of Coping and Anxiety/Depression	5–17	Hospital	Cross-sectional	The study highlights the significance of secondary control coping strategies in managing anxiety and depression in children with cancer, suggesting these strategies can be targeted in interventions for better emotional adjustment.	Interventions could enhance secondary control coping strategies to improve emotional outcomes for children with cancer.	Limited generalisability due to specific population and reliance on self- and parent reports for coping and distress.
45	Aeltsje Brinksma et al. 2014	Netherlands	Exploring the Response Shift Phenomenon in Childhood Patients with Cancer	2–17 years	Hospital	Retrospective pre- and post-test design	Response shift in overall HRQoL assessments, affecting the interpretation of change in HRQoL over time. Recalibration occurred in children’s perception of their initial health status.	Using specific tools like PedsQL that focus on multiple dimensions can reduce response shift effects in assessing HRQoL in paediatric oncology.	Exclusion criteria: insufficient command of Dutch language, children in palliative care; small sample size for generalisation. Incomplete data sets reduced the final sample size.
46	Lee Ai Chong et al. 2014	Malaysia	Clinical spectrum of children receiving palliative care in Malaysian Hospitals	0–18	Hospital	Observational cross-sectional study	A total of 315 patients were analysed, representing a variety of diagnostic categories. Notably, 50% underwent holistic needs assessments, with significant physical needs identified in those with nervous system diseases.	More education and training for paediatricians are imperative to improve quality care for all children, promote holistic assessments and advanced care planning.	Lack of local data on hospital-based paediatric palliative care; potential biases from volunteer study coordinators.
47	Wolfe et al. 2014	USA	Improving the Care of Children With Advanced Cancer by Using an Electronic Patient-Reported Feedback Intervention: Results From the PediQUEST Randomised Controlled Trial	2 years and older	Hospital	Parallel, multicenter pilot randomised controlled trial	Routine feedback did not significantly impact symptoms or HRQoL; however, improvements in emotional HRQoL were encouraging.	PRO feedback is valued and may contribute to care improvements; it could be considered further in younger children.	Limited impact on primary outcomes; feedback did not change symptoms or HRQoL significantly.
48	Joanne Wolfe, et al. 2014	USA	Improving the Care of Children With Advanced Cancer by Using an Electronic Patient-Reported Feedback Intervention: Results From the PediQUEST Randomised Controlled Trial	2 years and older	Hospital	Randomised Controlled Trial (RCT), pilot, multicenter	Routine PRO feedback did not significantly affect symptoms or overall QoL scores, but showed potential improvement in emotional QoL for certain age groups; satisfaction with feedback was high	PRO feedback interventions can aid in patient-centred care by improving the emotional well-being of children with cancer, especially beneficial in directing provider focus to psychosocial care	Limitations in statistical power, lack of blinding due to the study design, and varied duration of follow-up for participants
49	Danielle Cataudella, PsyD, et al. 2014	North America	Development of a Quality of Life Instrument for Children With Advanced Cancer: The Paediatric Advanced Care Quality of Life Scale (PAC-QoL)	Children and adolescents	Tool development study, no clinical or patient data collection setting involved.	Instrument development study, content validation	PAC-QoL includes items covering QoL domains identified as relevant, designed with parent and expert input for high relevance and clarity	PAC-QoL could provide a reliable way to assess QoL for children with advanced cancer and offer insights for tailored palliative care interventions	Further cognitive testing and psychometric evaluation needed

**Fig. 2:**
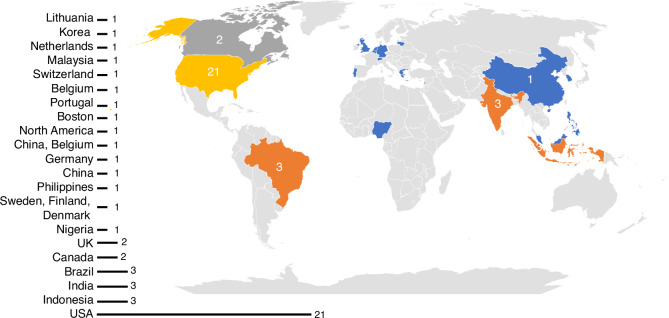
Geographic distribution of pediatric palliative care studies across countries. The world map illustrates the geographic distribution of included studies by country. The numbers within each country represent the total number of studies contributed. The highest number of studies originated from the United States (*n* = 21), followed by India (*n* = 3), Brazil (*n* = 3), and Indonesia (*n* = 3). Moderate contributions were observed from the United Kingdom (*n* = 2) and Canada (*n* = 2), while other countries including Lithuania, Korea, Netherlands, Malaysia, Switzerland, Belgium, Portugal, Germany, China, Philippines, Nigeria, and Nordic countries contributed one study each. Countries shaded in color indicate locations of included studies, whereas unshaded regions represent areas with no included studies.

The majority were published in the United States (*n* = 21) [2, 12, 27–37], followed by other countries [38–51] (Fig. 2).

**Fig. 3:**
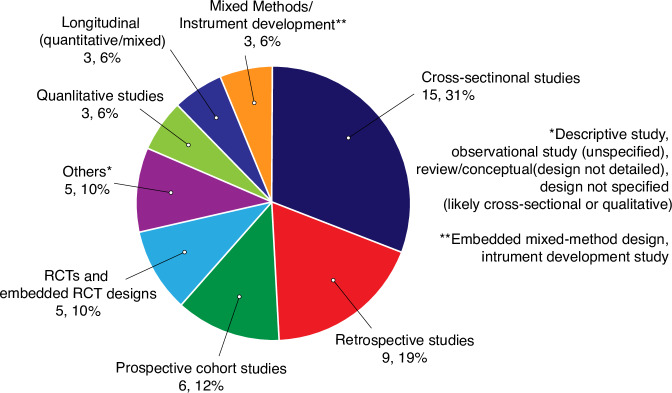
Distribution of included studies by study design. The figure presents the relative distribution of study designs among the included studies. Categories include cross-sectional, retrospective, prospective cohort, randomised controlled trials (including embedded designs), qualitative, longitudinal, mixed-methods or instrument development, and other study types. Percentages are shown for each category. “Others” includes descriptive, observational (unspecified), and studies with unclear or unspecified design.

Most studies employed cross sectional designs (*n* = 15) [12, 27, 28, 30–37, 40, 42–49], followed by retrospective studies (*n* = 9), prospective cohort studies (*n* = 6), and randomised controlled trials including embedded designs (*n* = 5). Other study designs accounted for (*n* = 5), while qualitative studies (*n* = 3), longitudinal studies (*n* = 3), and mixed-methods or instrument development studies (*n* = 3) were less frequently reported (Fig. 3) [2, 12, 27–51].

Although our inclusion criteria focused on studies involving children and adolescents aged 1–18 years, we observed that 4 studies [2, 34, 41, 50] included participants above 18 years of age, typically extending to young adults up to 25 years (with more than fifty percent less than 18 years of age, thus included) with various cancer diagnoses, as well as parents, caregivers, nurses and healthcare providers (Table 2).

**Table 2:** Patient characteristics in studies.

Study information		Participants & population			
S.no	Authors & year	Age range	Cancer type	Sample size	Family context
1	Murti Andriastuti et al. 2024	2–18 years old	solid tumours or blood cancer	269	Family involvement highlighted
2	Cowfer et al. 2024	5–17 years	Advanced cancer (relapsed or refractory)	20 children (90% female parents)	Most mothers participated
3	Cadamuro et al. 2024	2–18 years	Various cancers	157 participants (116 paediatric patients, 41 proxies)	Parents/caregivers involved
4	Grinde et al. 2024	2–18 years	CNS tumours, haematologic malignancies, non-CNS solid tumours	49	Caregivers provided proxy data for children aged 2–7
5	Hamner et al. 2024	5–18 years	Acute lymphoblastic leukaemia (ALL) and other cancers	43	Parental chronic stress evaluated
6	Wolfe et al. 2024	2–18 years	Advanced cancers (including brain tumours)	104	Parent and self-reports were collected
7	Sarah M et al. 2024	5–18 years	Various life-limiting and life-threatening conditions	10 children, 14 families, 31 participants total	Family’s emotional burden shared by healthcare professionals
8	Sharp et al. 2023	8–17 years	Cancer (various types)	255 (cancer group), 101 (comparison group)	Differences in SES between groups identified
9	Zhong et al. 2023	Not applicable	Not applicable	440 paediatricians	Not applicable
10	Joana Duran, DrNP, et al. 2021	8–17 years	Leukaemias/Lymphomas (42.4%), Brain/CNS tumours (27.3%), Sarcomas (24.2%), Other (6.1%).	33	Parents/legal guardians involved in providing consent and supporting at-home assessments.
11	Hendriks et al. 2021	≥18 years	All childhood cancers	28	Swiss childhood cancer survivors with long-term follow-up care
12	Ghoshal A et al. 2021	≤18 years	Paediatric oncology patients (majority with advanced stages of disease)	1135	Parents were primary caregivers In 97.9% of cases, many families had low incomes (<₹ 5000/month).
13	Erika Juškauskienė, RN et al.2021	5–12 years	Various oncological diagnoses (e.g. leukaemia, lymphomas, CNS tumours)	81	Family traditions and religious practices play a crucial role in children’s spiritual well-being.
14	Erica C. Kaye et al. 2021	2–18 years	Various oncological diagnoses (specific types not detailed)	321	Family dynamics and involvement are essential in palliative care planning and delivery.
15	Mitchell S, 2021	5–18 years	Life-limiting and life-threatening conditions (not specified)	31 participants (10 children, 14 families)	Participants included children and family members, highlighting family dynamics
16	Schneider A, Wurz A, Vogel A, 2021	4–18 years	Paediatric cancers	*n* = 130 dyads	Parent involvement
17	Snaman j et al. 2020	Not specified	Paediatric oncology patients	Not specified	Family dynamics significantly influence the delivery and effectiveness of palliative care.
18	Sung L, Zaoutis T, Ullrich CK, 2019	2–18 years	Paediatric Acute Myeloid Leukaemia (AML)	Not specified	Guardians provided proxy assessments for patients under 5 years; Self-report for patients over 5 years
19	Abby R. Rosenberg et al. 2016	2–18 years old	Various advanced cancers (leukaemia, solid tumours, etc.)	104 paediatric patients	Caregivers were primarily parents, with an emphasis on understanding family dynamics and sibling support
20	Tamires Vieira Carneiro, 2016	not specified	not specified	not specified	Involvement of family members in the care and support of paediatric oncology patients emphasised.
21	Momani T.G., Mandrell B.N., Gattuso J.S., et al. 2016	8–18 years	Acute Lymphoblastic Leukaemia (ALL)	150 participants	Participants included children and adolescents undergoing treatment for ALL, with family involvement considered in their responses
22	Lubis et al. 2015	5–18 years	Haematologic malignancies & solid tumours	46 children with cancer and 46 siblings	Control group consisted of normal siblings
23	Aeltsje Brinksma et al. 2014	2–17 years	Cancer	37 children, 80 parents	Child-parent pairs in paediatric oncology
24	Wolfe et al. 2014	2 years and older	Advanced Cance	104 (51 intervention, 53 control)	Not specified
25	Banayat, Campos, et al. 2024	Parents of children with cancer	Mixed cancer types, including solid tumours and haematologic cancer	156	Mostly indigent parents from various parts of the Philippines, often needing temporary housing near treatment centres
26	Nina Mogensen et al. 2024	1–18 years	Acute Lymphoblastic Leukaemia (ALL)	299	Socioeconomic factors, parent-reported toxicity
27	Nair et al. 2024	1–14 years	Metastatic Neuroblastoma	119	Family involvement in care decisions, some families refused treatment
28	Pangarso et al. 2024	Median age as a cutoff for younger vs. older children	Haematological tumours, solid tumours, rare tumours, brain tumours	49 children	Mostly poor families (based on hospital class); parents desired better communication and support
29	Brian T. Cheng, Tenzin Wangmo 2024	<18 years	Solid organ, brain, blood cancers	10,960 hospitalisations	Socioeconomic disparity in PC use
30	Kimberley Widger et al. 2024	0–18 years	Solid, CNS and haematologic cancers	572	Socioeconomic factors influenced SPPC
31	Silva, A.V.M.V. et al. 2023	1–18 years	Leukaemia, lymphoma	52	Family income < twice minimum wage; 76.9% mothers >8 years education
32	Yang Chen et al. 2022	2–18 years	Mixed cancer types including solid tumours and haematologic cancers	41 children	not specified
33	Trupti Narnaware, Maninderdeep Kaur, Sukhpal Kaur, Renu Madan 2022	4–18 years	Paediatric intracranial tumours	70	Children visiting OPD for post-treatment follow-up
34	Russell et al. 2021	8–18 years	Various paediatric cancers	87	Family psychosocial context (e.g. stress, structure, belief systems)
35	Song, I.G. et al. 2021	1–18 years	Paediatric cancer	109	NA
36	Robson, P. C., Dietrich, M. S., Akard, T. F. 2021	7–17 years	Haematologic malignancy	128	Family income $50,000 or less
37	Cheryl Rodgers et al. 2021	3–18 years	Acute lymphoblastic leukaemia (ALL)	327	Parent-proxy for younger children
38	Jennifer L. Raybin et al. 2021	3–17 years	All cancer types	98	Family-centred care, parent-proxy report
39	Nogueira A. et al. 2020	1–18 years,	leukaemia, brain tumours, or solid tumours.	72 eligible children	Family dynamics and support systems, including the involvement of caregivers, siblings and extended family in the child’s care.
40	Veronica Dussel et al. 2020	2 years and older	Advanced paediatric cancers	104 patients	High involvement of families in decision-making, with low-burden incentives and clear communication facilitating trust in study protocol
41	Marieke Van Schoors et al. 2019	0–18 years	Leukaemia, Non-Hodgkin Lymphoma	115 families (60 patients, 172 parents, 78 siblings)	All families Caucasian, living in Belgium, varied family sizes
42	Hamner, Latzman, Elkin, Majumdar 2018	6–18 years (Median age = 10.2 ± 3.6)	Various types, 40% with Acute Lymphoblastic Leukaemia (ALL)	43	Primarily biological parents; 79% mothers
43	Olagunju et al. 2016	7–12 years	Various paediatric cancers	72	Primarily parents (mostly mothers)
44	Veronica Dussel, MD, MPH, et al. 2015	2 years and older	Advanced cancer in paediatric patients.	231 (104 enroled)	Families involved in decision-making and participation surveys; incentives were provided.
45	Wolfe, J. 2015	2 years to 18	Advanced cancers (various types)	104 children	Parent reports used for younger children
46	Compas et al. 2014	5–17	Leukaemia, lymphoma, brain tumour, other solid tumours	334	Varied income levels
47	Lee Ai Chong et al. 2014	0–18	Various (Congenital malformations, nervous system diseases, neoplasms)	315	Siblings, caregivers
48	Joanne Wolfe et al. 2014	2 years and older	Children with advanced cancer where a decision not to pursue curative therapy was made	Intervention Group: 51; Control Group: 53	Children were accompanied by parents who participated in the surveys
49	Danielle Cataudella, PsyD, et al. 2014	Children and adolescents	Advanced malignancies	Bereaved parents (*n* = 8), HCPs (*n* = 7)	Included bereaved parents and healthcare professionals for content validation

Most studies were conducted in hospital-based settings (*n* = 24) [2, 12, 27, 29–32, 34–47, 49–51], with a few incorporating home-based care (*n* = 3) [28, 33, 48]. Studies addressed a broad range of outcomes, including symptom burden [12, 29–31, 35–37, 39, 43, 45, 46, 49, 51], quality of life [2, 27, 28, 30–32, 36, 38, 41–43, 49, 50], psychological well-being [2, 34], caregiver distress [32, 34], spiritual health [2, 34], communication quality [47] and end-of-life experiences [43, 44] (Table 3).

**Table 3:** Palliative care needs, quality of life studies and the relationship between them.

Study information			Palliative care needs					Quality of Life (QoL)			Relationship between palliative care needs & QoL	
S. no	Authors & year	Study title	Physical needs	Emotional needs	Social needs	Spiritual needs	Other needs	QoL domains	QoL measurement tool	QoL scores/findings	Association found	Key insights
1	Murti Andriastuti et al. 2024	Family functioning, parental cancer-related emotions, and quality of life in childhood cancer patients	None were explicitly mentioned in the study.	Significant associations were found between low quality of life (QoL) scores and parental emotions such as loneliness, helplessness, and uncertainty (*P* < 0.001).	The study highlighted family cohesion and adaptability as key factors in the emotional well-being of both children and parents.	Not directly addressed in this study.	Not covered	Emotional, social and family functioning	quality of life (PedsQL), (2) family function (FACES III) and (3) family cancer-related emotions(SSERQ)	Children aged 8-12 years had lower PedsQL scores, solid tumours correlated with worse QoL and connected families showed higher scores, while negative parental emotions linked to lower QoL in children	Family functioning & parental emotions linked to QoL	Family dynamics and parental stress affect QoL
2	Banayat, Campos, et al. 2024	Care Needs of Parents of Children with Cancer	Needs like rest and management of fatigue	High emotional distress due to the child’s illness and treatment.	There is a need for support groups and communication with other parents in similar situations.	Prayer was used for coping and acceptance.	Financial Needs, Temporary Housing, Access to treatment	Emotional, Spiritual	Revised Cancer Patient Needs Assessment: Parent Perspective (rCPNQ)	No QoL Scores provided	Significant association between care needs and variables like tumour diagnosis, surgery and time since diagnosis.	Financial needs were the most critical, followed by access to temporary housing and spiritual coping mechanisms.
3	Cowfer et al. 2024	Relationships Between Parental Anxiety and Child Quality of Life in Advanced Childhood Cancer	Not explicitly stated	Assessed indirectly	Not explicitly stated	Not explicitly stated	Not explicitly stated	HRQoL (PedsQL Generic and PedsQL Cancer)	STAI-T for parental anxiety, PedsQL for HRQoL	Significant inverse correlations between parental anxiety and child psychosocial HRQoL (e.g. rs = –0.54 for psychosocial HRQoL).	Inverse associations between parental anxiety and parent-reported child psychosocial HRQoL. Positive but not significant correlations with child-reported HRQoL.	Parent anxiety inversely affects child psychosocial quality of life, highlighting the need for parental mental health support
4	Cadamuro et al. 2024	Association between multiple symptoms and quality of life of paediatric patients with cancer in Brazil	Not explicitly stated	Assessed indirectly	Not explicitly stated	Not explicitly stated	Not explicitly stated	Overall QoL (PedsQL)	PedsQL and SSPedi-BR tools	A high prevalence of symptoms was observed, with fatigue (62.4%) and changes in taste (56.7%) being the most common. Symptoms negatively influenced QoL.	Significant negative influence of all symptoms on QoL, particularly in emotional domains.	Symptoms experienced during treatment were prevalent and significantly impacted QoL.
5	Grinde et al. 2024	Symptom Adverse Events and Quality of Life of Children With Advanced Cancer: Results From a Longitudinal Study	Pain, nausea, insomnia, hot flashes, fatigue	Anxiety, treatment anxiety, worry	Cognitive problems, perceived physical appearance, communication	N/A	N/A	Pain, nausea, procedural anxiety, cognitive problems	Paediatric Quality of Life Inventory (PedsQL™) Cancer Module 3.0	Mean total score: 73.86; CNS tumour patients reported lower QoL	Negative association between symptom AEs (e.g. nausea, anxiety, fatigue) and QoL	Time-specific significant differences: poorer QoL observed with symptom AEs at Week 8
6	Hamner et al. 2024	Quality of Life Among Paediatric Patients With Cancer: Contributions of Time Since Diagnosis and Parental Chronic Stress	Hurts, aches, and physical functioning	Worrying about the future, emotional functioning	Getting along with other children, social functioning	N/A	School-related functioning	Emotional, physical, school and social functioning	Paediatric Quality of Life Inventory (PedsQL)	TSD positively associated with physical functioning (*r* = 0.30); parental stress negatively associated with emotional (*r* = −0.54), physical (*r* = −0.41) and social (*r* = −0.44) functioning	Parental chronic stress contributed incrementally to reduced physical, emotional and social functioning across paediatric cancer patients	Parental chronic stress negatively impacts QoL across emotional, physical and social domains in paediatric patients
7	Wolfe et al. 2024	Symptoms and Distress in Children With Advanced Cancer: Prospective Patient-Reported Outcomes From the PediQUEST Study	Pain (48%), fatigue (46%), drowsiness (39%)	Pain (48%), fatigue (46%), drowsiness (39%)	Emotional and social support needs are not specified	N/A	High symptom burden	Physical, psychological symptom distress; total symptom burden	PQ-MSAS (PediQUEST Memorial Symptom Assessment Scale)	Pain was prevalent (62%) in the last 12 weeks of life, with high distress levels (58%); distress worsened with disease progression or high-intensity treatment	High-intensity cancer therapy and disease progression correlated with greater symptom distress; mild cancer-directed therapy improved psychological distress in the final weeks	Children with advanced cancer endure substantial symptom distress, particularly as the disease progresses and treatment intensity increases
8	M Sarah et al. 2024	Experiences of healthcare, including palliative care, of children with life-limiting and life-threatening conditions and their families: a longitudinal qualitative investigation	Access to palliative care is dependent on the availability of specialist services	Emotional burden due to fragmented care and a lack of discussion on death	Interpersonal relationships with healthcare professionals were vital	Not mentioned	Other priorities for living expressed by children beyond their condition	Not specified	Not specified	Not specified	Lack of integration of palliative care with broader healthcare	Trusted relationships with healthcare professionals are crucial; care often feels fragmented
9	Nina Mogensen et al. 2024	Quality of life in children and adolescents after treatment for acute lymphoblastic leukaemia	Physical functioning, as measured using the PedsQL, showed lower scores in high-risk (HR) patients, particularly for School Functioning. Parent-reported toxicity during treatment significantly influenced physical quality of life (HRQoL).	Assessed	Assessed	Not specified	None	Physical, Emotional, Social, School	Paediatric Quality of Life Inventory (PedsQL) version 4.0	Older children (8 years and older) reported similar HRQoL compared to the reference population, except for lower scores for School Functioning in HR patients. Parent-proxy reports for children aged 5–7 years were notably lower than the reference population.	Parent-reported toxicity was a significant predictor of lower HRQoL scores, particularly in the physical and total scores.	The most critical factor affecting HRQoL after ALL treatment was parent-reported toxicity during treatment, emphasising the need to minimise complications during treatment.
10	Nair et al. 2024	Optimising Palliative Care for Children with Metastatic Neuroblastoma and the Paediatrician’s Role in a Shared Care Model	Pain, nausea, vomiting, constipation, abdominal distension, bony swellings, proptosis	Psychosocial support by oncology & PC team, emotional distress linked to severe pain	Support from oncology & PC team, local paediatricians, and family involvement	Psychosocial support offered, not explicitly detailed	Parental refusal for treatment, treatment shift to PC, end-of-life needs	Symptom burden management, care transition from oncology to PC, family support during relapse and end-of-life stages	No specific QoL tool mentioned, focus on symptom management and pain relief	Higher pain and symptom burden at relapse and end-of-life stages	Integration of palliative care early during metastatic NB can reduce symptom burden and improve QoL for paediatric patients	Early referral to palliative care, shared care model between oncology and PC teams, and inclusion of local paediatricians can optimise care
11	Pangarso et al. 2024	Discovering Needs for Palliative Care in Children with Cancer in Indonesia	Breathing difficulties (82%), pain (80%), appetite loss (80%)	Depression (46% no intervention), anxiety (45% no intervention), sadness (33% no intervention)	Loneliness; desired more interaction with school (71%), friends (63%), family (57%)	inferred through family decision-making and preferences about treatment at the end of life	Effective communication between doctors and families; informed decision-making regarding treatment goals	Physical, emotional and social well-being	Semi-structured questionnaire designed and pilot-tested for cultural relevance	qualitative findings highlight unmet needs in multiple domains	Quality of life is negatively affected by inadequate symptom management, emotional neglect and limited social support	Enhancing symptom management, emotional support, and family involvement improves the quality of life for children with cancer
12	Brian T. Cheng, Tenzin Wangmo 2024	Palliative care utilisation in hospitalised children with cancer	Limited focus; inferred from QoL improvement	linked to QoL	Socioeconomic disparity in PC access	Not stated	Lower healthcare costs with PC use	Increased QoL with PC use	Not mentioned	Not mentioned	PC linked to lower costs, shorter stays	1 in 20 paediatric inpatients with high mortality risk received PC. Utilisation varied by socioeconomic factors. Older age and solid cancers were predictors of PC use.
13	Kimberley Widger et al. 2024	Predictors of Specialised Paediatric Palliative Care Involvement and Impact on Patterns of End-of-Life Care in Children With Cancer	Lowered intensity care needs at EOL	Reduced stress associated with SPPC	Socioeconomic disparities in care access	Not mentioned	Uneven access to SPPC	Not measured	Not mentioned	Not reported	SPPC reduced odds of ICU admission by 80%	SPPC led to significantly less intensive EOL care compared to general or no palliative care. Access was influenced by socioeconomic and geographic factors.
14	Sharp et al. 2023	Predictors of psychological functioning in children with cancer: disposition and cumulative life stressors	N/A	Lower anxiety in the cancer group compared to healthy peers	N/A	N/A	N/A	Depression, Anxiety, PTSS	Children’s Depression Inventory (CDI), Screen for Child Anxiety Related Emotional Disorders (SCARED), UCLA PTSD Reaction Index	No significant differences in depression and PTSS; lower anxiety in cancer group (*p* < 0.01). Clinically elevated symptoms: 6.3% cancer group, 6.9% control (depression); 27.8% cancer group, 35.6% control (anxiety); PTSS clinically elevated: 11.1% cancer group, 13.9% control.	Psychological functioning is primarily predicted by disposition, with secondary influence from stressful life events.	Children with cancer are generally resilient; factors influencing adjustment are like those without serious illness.
15	Zhong et al. 2023	Chinese and Belgian paediatricians’ perspectives toward paediatric palliative care	Not directly mentioned	Not directly mentioned	Paediatricians’ support systems at work	Not directly mentioned	Personal obstacles (e.g. trauma from child’s death)	Not applicable	Not applicable	Not applicable	Yes	Chinese paediatricians face more barriers due to a lack of unit support, and experience positively influences perspectives
16	Silva, A.V.M.V. et al. 2023	Impact of childhood cancer on health-related quality of life in children and adolescents: self-perception and mother’s perception	NA	Procedural anxiety, Nausea, Concern	NA	NA	NA	Pain, Nausea, Procedural Anxiety, Treatment Anxiety, Worry, Cognitive Problems, Physical Appearance, Communication	Paediatric Quality of Life Inventory (PedsQL™) 3.0 Cancer Module	Lower scores in the Procedural Anxiety, Nausea and Concern domains; no associations were found in mothers’ perceptions, but self-reports showed significant associations.	Age group (9-18 years) and diagnosis of lymphoma negatively influenced self-perception of HRQoL	The child/adolescent’s age, type of cancer and mother’s education level negatively impacted HRQoL self-perception, indicating a gap between children’s and mothers’ perceptions.
17	Yang Chen et al. 2022	Palliative sedation for children at the end of life: a retrospective cohort study	Complex symptoms, pain management	Anxiety, depression	Family involvement	not specified	not specified	not specified	not specified	Overall symptom relief: Group A 100% vs Group B 66.7% (*p* = 0.041); survival time longer in Group A (*p* = 0.047)	Palliative sedation controls symptoms and does not shorten hospitalisation	Palliative sedation is effective for symptom control in complex cases
18	Trupti Narnaware, Maninderdeep Kaur, Sukhpal Kaur, Renu Madan 2022	Assessment of Quality of Life and Long-term Health-related Problems Among Children with Intracranial Tumour	Fatigue (57.1%), weakness (41.4%), headache (25.7%), muscle power issues	Anxiety, emotional health issues	School functioning issues	N/A	Neurocognitive dysfunction (attention, thinking, reading), endocrine problems	Physical health, emotional health, social activities and school functioning	PedsQL TM Generic Core 4.0 Scale	Total QoL score was 70.8 ± 24.6; the most affected domain was school functioning, followed by physical functioning	Long-term health problems (fatigue, neurocognitive dysfunction, etc.) impact QoL	Children with intracranial tumours show average HRQoL and are at risk for long-term health-related issues impacting several life domains
19	Joana Duran, DrNP, et al. 2021	Quality of Life and Pain Experienced by Children and Adolescents with Cancer at Home Following Discharge	Emotional functioning is worse for females and adolescents, anxiety related to treatment, worries	Social functioning is lower in sarcoma patients	Not addressed	None	Physical, emotional, cognitive and social.	Paediatric Quality of Life Inventory (PedsQL™ Cancer Module, Multidimensional Fatigue Scale).	Pain and fatigue are linked to lower QoL scores, especially in physical and emotional domains; sarcoma patients reported lower cognitive and social functioning.	Yes, pain and fatigue were associated with lower QoL, especially physical and emotional functioning.	Pain and fatigue significantly impact QoL at home, especially in physical and emotional functioning. Variations by age, gender and diagnosis.	NA
20	Hendriks et al. 2021	The unmet needs of childhood cancer survivors in long-term follow-up care	NA	Lacking psychosocial support	Lacking collaboration and decentralisation of care	N/A	Personalised and specialised services	N/A	Semistructured interviews	Not directly measured	Yes	Need for psychosocial support and personalised, centralised follow-up care for survivors
21	Ghoshal A et al. 2021	Specialist Paediatric Palliative Care Referral Practices in Paediatric Oncology: A Large 5-Year Retrospective Audit	54.2% of patients had high symptom burden at referral; 10.5% required palliative radiotherapy.	30.3% of referrals were made for counselling and communication, indicating a high level of emotional needs.	Most referrals were late, and families frequently lacked access to local paediatric healthcare services.	Not specifically detailed; the focus was on practical end-of-life care.	Most patients were cared for at home by local general practitioners (90.8%).	The main focus was on symptom burden and communication needs at referral; late referrals were common, and liaison with local GPs was essential.	Retrospective review of electronic medical records and audit of inpatient and outpatient notes.	70.6% of deaths were anticipated; 84.6% of consultations occurred in outpatient settings. Only 4.9% of patients had more than two follow-ups after referral.	Late referrals were common; there was a significant focus on communication and symptom management. However, only 21.2% of patients had access to local paediatric health services.	Oncologists referred paediatric patients late in the disease trajectory. Many referrals were for counselling, but patients had high symptom burden at the time of referral.
22	Erika Juškauskienė, RN et al. 2021	Spiritual Well-Being and Related Factors in Children With Cancer	Physical pain assessment and management were essential.	Emotional well-being is closely tied to spiritual health; children often express their feelings and happiness in significant ways.	Family support and church attendance impact spiritual well-being.	Children demonstrated varying levels of spiritual well-being, with the importance of familial and community traditions being noted.	Not specified.	Key domains include physical health, emotional state, social connections and spiritual well-being, which influence overall QoL.	Feeling Good, Living Life; Oxford Happiness Questionnaire; WHO-5; PedsQL™3.0 Cancer Module; Wong-Baker FACES® Pain Rating Scale	High scores in communal and personal spiritual well-being; lower in transcendental domains. Happiness has a significant impact on spiritual well-being across various domains.	Age, education level and family composition showed associations with spiritual health and happiness.	Importance of family traditions and church attendance in enhancing spiritual well-being; children familiar with religious practices despite their young age.
23	Erica C. Kaye et al. 2021	Illness and End-of-Life Experiences of Children with Cancer Who Receive Palliative Care	High burden of intensive treatments; many patients experienced mechanical ventilation and resuscitation	Emotional distress associated with intensive treatments; need for psychological support highlighted.	Family involvement in care decisions and emotional support is crucial during end-of-life care.	Not explicitly addressed in the abstract.	Not specified.	Key domains include medical treatment burden, emotional distress, family involvement and care setting (ICU vs. home/hospice).	Not specified.	79.4% received experimental therapy; 40.5% on phase I trials; 35.5% received cancer-directed therapy in the last month; late PC involvement correlated with ICU death.	Late PC involvement (less than 30 days before death) increased odds of dying in the ICU compared to earlier involvement (OR: 4.7).	Children receiving palliative care often endure extensive treatments; timely involvement of palliative care can significantly impact end-of-life experiences and care settings.
24	Mitchell S, 2021	Experiences of healthcare, including palliative care, of children with life-limiting and life-threatening conditions and their families: a longitudinal qualitative investigation	Fragmented healthcare system, need for physical care coordination	Emotional acceptance of conditions, focus on living	Need for social support, family dynamics	Rarely discussed existential/spiritual concerns	Importance of trusted relationships with professionals	Health-related quality of life, emotional well-being	Thematic analysis of qualitative interviews	Insights into experiences and unmet needs of children and families	Children accepted conditions as part of life; systemic barriers to care exist	Highlights the need for a child and family-centred approach to integrate palliative care into healthcare services
25	Schneider A, Wurz A, Vogel A, 2021	Illness perceptions, fear of progression, and health-related quality of life during acute treatment and follow-up care in paediatric cancer patients and their parents: a cross-sectional study	Yes	Yes	Not specified	Not specified	Not specified	Physical well-being, emotional well-being, self-esteem, family, friends, daily functioning, illness	KINDL-R	In acute treatment, patient perceptions explained variation in HRQoL; in follow-up care, FoP and parent’s perceptions explained additional variation.	Illness perceptions and FoP significantly impact HRQoL in paediatric cancer patients and their parents, particularly in follow-up care.	Emphasises the importance of psychological factors on well-being, especially in follow-up care; recommends screening for illness perceptions and FoP and developing family-focused interventions.
26	Russell et al. 2021	Psychosocial risk, symptom burden, and concerns in families affected by childhood cancer	Pain, tiredness, nausea, drowsiness, appetite	Depression, anxiety, distress, well-being issues	Family/social relationships impacted, lack of support	Emotional and practical concerns over illness	Informational needs, family structure and understanding treatment	Symptoms: physical, emotional, social; concerns related to diagnosis, treatment trajectory	Psychosocial Assessment Tool (PATrev), ESAS-r, Canadian Problem Checklist (CPC)	Higher family psychosocial risk was associated with higher symptom burden and more concerns	Families with high psychosocial risk reported greater symptom burden and concerns, suggesting the need for resource prioritisation based on psychosocial risk levels	Psychosocial risks and symptom burden should be evaluated continuously throughout treatment; need for targeted interventions based on risk and ongoing concerns
27	Song, I.G. et al. 2021	Paediatric palliative screening scale as a useful tool for clinicians’ assessment of palliative care needs of paediatric patients	Symptom burden	Toxicities of therapy	NA	NA	NA	Trajectory of disease, symptom burden, treatment preferences, expected outcomes and estimated life expectancy	Paediatric Palliative Screening Scale (PaPaS)	The mean PaPaS score is higher in the PPC team; scores are above the cut-off for referral in both groups. PaPaS correlated with the Lansky performance scale.	Good agreement between primary care and PPC teams for PaPaS scores.	The PaPaS is effective for assessing palliative care needs in paediatric patients and assists in the timely identification of palliative care.
28	Robson, P. C., Dietrich, M. S., Akard, T. F. 2021	Associations of Age, Gender, and Family Income with Quality of Life in Children With Advanced Cancer	Not specified	Procedural anxiety, treatment anxiety	Not specified	Not specified	Not specified	Pain, nausea, procedural anxiety, treatment anxiety, worry, cognitive problems, perceived physical appearance and communication	Paediatric Quality of Life Inventory (PedsQL) Cancer Module	Statistically significant positive associations of age and income with PedsQL scores (*p* < 0.05), but not with gender (*p* > 0.05). Strongest correlations for age with procedural anxiety (beta = 0.42) and treatment anxiety (beta = 0.26). Positive correlation between family income levels and PedsQL scores (*R* = 0.49).	Age and income significantly impact QOL, with younger children and those from lower-income families at higher risk for poorer QOL.	Oncology nurses should identify families who may benefit from additional resources to promote QOL.
29	Cheryl Rodgers et al. 2021	Childhood Cancer Symptom Cluster: Leukaemia and Health-Related Quality of Life	Fatigue, sleep disturbance, pain, nausea	Depression, emotional challenges	Challenges due to treatment impact	Not specified	None specified	Physical, psychological	HRQOL questionnaire (self-reported or proxy)	Mean HRQOL significantly increased over time (*p* < 0.001), with a negative association between symptom severity and HRQOL at both post-induction and maintenance therapy start.	Negative association between Childhood Cancer Symptom Cluster - Leukaemia (CCSC-L) and HRQOL scores (*β* = −0.53 at post-induction, *β* = −0.33 at maintenance therapy start) and lower HRQOL in participants with more severe CCSC-L symptoms (*β* = −0.42)	Symptoms such as fatigue, sleep disturbance, pain, nausea and depression are interrelated and impact HRQOL in children with leukaemia undergoing ALL therapy.
30	Jennifer L. Raybin et al. 2021	Associations between demographics and quality of life in the first year of cancer treatment	Addressed through posture assessment (e.g. thoracic kyphosis)	Emotional distress measured via Faces Scale, incl. worry and anxiety	Not specified	Not specified	Demographic Needs	Physical, Emotional, Cognitive, Social	PedsQL 3.0 Cancer Module, Faces Scale, Posture Measure	Older age is associated with lower QoL and higher worry, pain and nausea scores	Older age correlated with lower QoL scores; worry and posture were impacted by age.	Targeted interventions can focus on managing worry, pain and nausea, especially in older children.
31	Snaman J et al. 2020	Paediatric Palliative Care in Oncology	Comprehensive physical assessments are essential; management of symptoms is critical.	Emotional support is vital for both patients and families, focusing on coping strategies.	Social support from family and friends enhances the quality of life; integration of services is important.	Spiritual care should be tailored to individual beliefs and practices.	Transition support from treatment to palliative care is crucial.	Key QoL domains include physical, emotional and social well-being; spiritual well-being also plays a role.	Not specified; likely varies by study component.	Findings highlight the importance of a multidisciplinary approach to palliative care in improving the QoL of paediatric oncology patients. Emotional support has significant positive impacts.	There is a strong association between the timing of palliative care referral and the QoL outcomes; early intervention is critical.	The study emphasises the importance of integrating palliative care into standard oncology practices, providing holistic support to paediatric patients and their families.
32	Nogueira A. et al. 2020	The needs of children receiving end of life care and the impact of a paediatric palliative care team	Specific physical needs related to CCC, including symptom management, pain relief, and nutritional support.	Psychological support for both children and families, including counselling and mental health resources to address grief and anxiety.	Support for social integration, family dynamics, and peer relationships, focusing on maintaining connections with friends and family during illness.	Spiritual care addressing existential questions, providing opportunities for spiritual practices, and support for families’ spiritual beliefs and rituals.	Additional needs that may arise, such as financial support, educational resources and respite care for families.	Domains of quality of life assessed could include physical health, emotional well-being, social functioning and spiritual health.	Potential tools include validated questionnaires like the Paediatric Quality of Life Inventory (PedsQL) or the Childhood Quality of Life Questionnaire (ChQoL).	Specific scores indicating levels of quality of life, potentially reported as mean scores or changes in scores pre- and post-intervention	Analysis showing the relationship between palliative care needs and quality of life; potential positive correlation indicating that better palliative care may enhance QoL.	Insights drawn from the study regarding the multifaceted needs of children with CCC and the importance of addressing these needs to improve overall quality of life.
33	Veronica Dussel et al. 2020	Feasibility of Conducting a Palliative Care Randomised Controlled Trial in Children With Advanced Cancer: Assessment of the PediQUEST Study	Addressed through symptom assessment tools; electronic self-reports enabled pain and discomfort monitoring	Anxiety about symptoms, treatment	Support for families provided, incentives for participation	Not specified	Altruism	Physical, emotional, social	Electronic patient-reported outcomes (PediQUEST) system	87 completed study, 24 died, 17 dropped out; high self-report rate of 94% among child/teen respondents; attrition high near end of life (60%)	Attrition higher in advanced cases with brain tumours; lower for those completing up to 3-month follow-up	Retention adequate; families enroled to help others, appreciated low-burden procedures; helpful staff attitudes contributed to enrolment
34	Sung L, Zaoutis T, Ullrich CK, 2019	Quality of Life in Paediatric Acute Myeloid Leukaemia: Report from the Children’s Oncology Group	Pain, fatigue	Anxiety, worry	Communication issues	N/A	Cognitive problems	Pain and hurt, nausea, procedural and treatment anxiety, cognitive problems, perceived physical appearance, communication	PedsQL 4.0 Generic Core Scales, PedsQL 3.0 Acute Cancer Module, PedsQL Multidimensional Fatigue Scale	Higher scores indicate better QoL; findings showed decreased QoL during intensive chemotherapy cycles	Association between chemotherapy and reduced QoL	Chemotherapy cycles were associated with significant decreases in QoL domains such as physical and emotional well-being
35	Marieke Van Schoors et al. 2019	Family Members Dealing With Childhood Cancer: A Study on the Role of Family Functioning and Cancer Appraisal	Not specified	Significant focus on cancer-related emotional reactions (loneliness, uncertainty, helplessness)	Family cohesion and conflict, expressiveness within the family	Not specified	Cancer-related stress, family dynamics	Emotional well-being, family support	Paediatric Quality of Life Inventory (PedsQL), Family Environment Scale (FES), Situation-Specific Emotional Reactions Questionnaire (SSERQ), Maudsley Marital Questionnaire (MMQ)	Not specified, variations in QoL among patients, parents and siblings	Association found between family functioning, cancer appraisal and QoL	Differences in family members’ perceptions highlight the need for individual and family-level intervention
36	Hamner, Latzman, Elkin, Majumdar 2018	Quality of Life Among Paediatric Patients With Cancer: Contributions of Time Since Diagnosis and Parental Chronic Stress	Higher TSD is linked to improved physical functioning; parental stress linked to poorer physical functioning	Parental stress is associated with poorer emotional functioning	Parental stress is associated with poorer social functioning	Not assessed	Not assessed	Physical, Emotional, Social, School	Paediatric Quality of Life Inventory (PedsQL)	Higher scores indicate better QoL (reverse-coded for analysis); TSD positively correlated with physical functioning; parental stress negatively correlated with emotional, social and physical functioning	Positive association between TSD and physical functioning; negative association between parental stress and physical, emotional and social functioning	Parental chronic stress is a strong predictor of reduced QoL, particularly affecting emotional and social aspects beyond physical health
37	Abby R. Rosenberg et al. 2016	Quality of Life in Children With Advanced Cancer: A Report From the PediQUEST Study	Pain, fatigue, sleep issues	Anxiety, sadness, and coping challenges for both the children and their parents	Need for strong support networks; the burden on family caregivers, sibling support	Spiritual distress and existential concerns, particularly in cases where disease progression was severe	Communication needs between the family and the healthcare team regarding treatment and prognosis	Physical, emotional and social well-being	PediQUEST quality of life survey, child self-report and parent proxy report	Decreasing quality of life as cancer progresses, particularly in the emotional and physical domains; both children and parents reported lower QoL near end-of-life stages	Strong association between unmet palliative care needs (especially emotional and physical) and lower QoL	Addressing emotional distress and physical symptoms like pain early on can significantly improve QoL
38	Tamires Vieira Carneiro, 2016	Quality of Life of Paediatric Oncology Patients	Pain management, symptom relief, and nutritional support specific to paediatric oncology patients.	Coping with diagnosis, anxiety about treatment outcomes, and emotional support for patients and families.	Family support networks, peer support, and access to social services.	Consideration of spiritual concerns and existential questions related to illness and treatment.	Access to multidisciplinary care and coordination among healthcare providers.	The physical, emotional, social and spiritual domains that impact the lives of paediatric oncology patients.	QoL assessment tools	Key findings on QoL outcomes related to paediatric oncology are not specified; should focus on how treatment impacts QoL.	Potential associations between unmet palliative care needs and decreased QoL outcomes in paediatric oncology patients.	Importance of addressing all dimensions of care to improve overall QoL for paediatric oncology patients.
39	Momani T.G., Mandrell B.N., Gattuso J.S., et al. 2016	Children’s Perspective on Health-Related Quality of Life during Active Treatment for Acute Lymphoblastic Leukaemia: An Advanced Content Analysis Approach	Symptom management during treatment	Emotional impact of cancer and treatment	Focus on social interactions, especially friendships	Spirituality is not directly explored	Changes in self-perception during illness	Health-related quality of life	Two validated interview questions	HRQoL affected by symptoms and treatment milestones	Age, gender, risk group and time in treatment impacted code frequencies	Adolescents focused more on friendships, while females reported more negative experiences. Adolescents also reported positive life changes due to illness.
40	Olagunju et al. 2016	Child’s symptom burden and depressive symptoms among caregivers of children with cancers: an argument for early integration of paediatric palliative care	Pain, nausea, lack of energy	Depression, worry	Community support	Spiritual beliefs	Not specified	Memorial Symptom Assessment Scale (MSAS)	Memorial Symptom Assessment Scale (MSAS)	38.2% of caregivers screened positive for depression; common symptoms include pain (≥50%) and lack of energy (68%)	Positive correlation (*r* = 0.58, *P* < 0.05) between the child’s symptom burden and caregiver depression	Awareness of childhood cancers is crucial; integrating palliative care early can benefit caregivers and children.
41	Lubis et al. 2015	Quality of life in children with cancer and their normal siblings	Assessed via physical function in PedsQL	Assessed via emotional function in PedsQL	Assessed via social function in PedsQL	Not explicitly stated	Assessed via school function in PedsQL	Physical, emotional, social and school functions (PedsQL 4.0)	PedsQL 3.0 cancer module & PedsQL 4.0 generic scales	Children with cancer had significantly lower scores in all domains compared to their normal siblings. Physical function (36.9 vs. 80.7), emotional function (40.4 vs. 69.3), social function (71.5 vs. 93.9), school function (20.7 vs. 74.2) and total score (42.1 vs. 79.3), all *P* = 0.0001.	QoL was significantly lower in children with cancer compared to their siblings, especially in school function. No significant differences were found between children with haematologic malignancies and those with solid tumours.	The study found that cancer treatment affects multiple domains of QoL, with school function being the most impacted. The emotional function was also notably lower in children with cancer.
42	Veronica Dussel, MD, MPH, et al. 2015	Feasibility of Conducting a Palliative Care Randomised Controlled Trial in Children With Advanced Cancer: Assessment of the PediQUEST Study	No direct physical needs measured, but attrition during physical decline was a key consideration.	Emotional support for children and teenagers, as well as their families; reasons for participating include altruism and staff support.	Not explicitly studied; however, the role of caregivers in participation was emphasised.	Not addressed	Participation burden (e.g. survey completion, follow-up attrition)	Physical, emotional, cognitive, social and overall well-being.	Paediatric Quality of Life Inventory (PedsQL™) and PediQUEST surveys.	87 patients completed, with median intermittent attrition (IA) of 41% in the first 20 weeks and over 60% in the 8 weeks before death. Post-inclusion retention was adequate	Yes, emotional needs and altruistic motivations influenced participation; IA increased near end of life, highlighting the challenge of late-stage care.	Study demonstrated feasibility of PPC-RCT; many families participated for altruistic reasons. Recruitment could increase by reducing follow-up duration.
43	Wolfe, J. 2015	Symptoms and Distress in Children With Advanced Cancer: Prospective Patient-Reported Outcomes From the PediQUEST Study	Pain, fatigue, drowsiness	Irritability, psychological distress (especially near the end of life)	Not specifically addressed	Not specifically addressed	Not specifically addressed	Physical and psychological symptoms	PediQUEST Memorial Symptom Assessment Scale (PQ-MSAS)	High distress was reported, especially in pain (62%) and irritability during the last 12 weeks of life	Higher distress scores are associated with factors such as female gender, brain tumours, recent disease progression and high-intensity treatments	Children with advanced cancer experience significant symptom distress, necessitating intensive symptom management
44	Compas et al. 2014	Children and Adolescents Coping With Cancer: Self- and Parent Reports of Coping and Anxiety/Depression	N/A	Yes	N/A	N/A	N/A	Anxiety/Depression	Youth Self-Report (YSR) and Child Behaviour Checklist (CBCL)	N/A	Yes	Secondary control coping is important for adjustment in children during cancer treatment.
45	Aeltsje Brinksma et al. 2014	Exploring the Response Shift Phenomenon in Childhood Patients with Cancer	Not specified	Not specified	Not specified	Not specified	Overall HRQoL	PedsQL 4.0 Generic Core Scale, Cantril’s ladder	Children and parents experienced a response shift in overall HRQoL; no differences found between PedsQL then- and pretests.	Children and parents experienced a response shift in overall HRQoL; no differences found between PedsQL then- and pretests.	Yes, response shift in overall HRQoL ratings (Cantril’s ladder) led to underestimating improvements.	Knowledge of response shift can aid in better interpretation of HRQoL outcomes in paediatric cancer care.
46	Lee Ai Chong et al. 2014	Clinical spectrum of children receiving palliative care in Malaysian Hospitals	Yes	Yes	Yes	Yes	Various	various	Not specified	Not specified	Not specified	Awareness of paediatric palliative care has led to paediatrician-led services; patients have varied diagnoses and needs; further education for paediatricians is necessary.
47	Wolfe et al. 2014	Improving the Care of Children With Advanced Cancer by Using an Electronic Patient-Reported Feedback Intervention: Results From the PediQUEST Randomised Controlled Trial	Seattle Children’s Hospital	Improved emotional HRQoL (Δ8.1; 95% CI, 1.8–14.4)	Not specified	Not specified	Health-related quality of life	Paediatric Quality of Life and Evaluation of Symptoms Technology (PediQUEST), Memorial Symptom Assessment Scale (MSAS), Paediatric Quality of Life Inventory 4.0 Generic Core Scales (PedsQL4.0)	MSAS, PedsQL4.0	Subgroup analysis showed improvements in emotional HRQoL.	Feedback improved emotional and Sickness scores in children aged ≤8 years who survived 20 weeks	Routine feedback of PROs was valued by children, parents and providers, contributing to the effectiveness of psychosocial consults.
48	Joanne Wolfe, et al. 2014	Improving the Care of Children With Advanced Cancer by Using an Electronic Patient-Reported Feedback Intervention: Results From the PediQUEST Randomised Controlled Trial	Physical symptoms tracked with MSAS and PediQUEST reports	Improved emotional QoL observed in subgroup analyses among children ≥8 years	Not specified	Not specified	Not specified	Physical, Emotional	Paediatric Quality of Life Inventory 4.0 Generic Core Scales (PedsQL4.0), Memorial Symptom Assessment Scale (MSAS)	Emotional QoL improved (PedsQL4.0) in children ≥8 years; No significant effect on MSAS trends	PRO feedback improved emotional QoL for children ≥8 years who survived 20 weeks	Routine feedback of patient-reported outcomes (PROs) is valued by patients, families and providers and encourages provider action for psychosocial support in 56% of cases
49	Danielle Cataudella, PsyD, et al. 2014	Development of a Quality of Life Instrument for Children With Advanced Cancer: The Paediatric Advanced Care Quality of Life Scale (PAC-QoL)	Not specified	Emotional distress, sense of well-being	Social interactions with family, caregivers	No specific	Age-appropriate needs, sensitivity of items	Age-appropriate needs, sensitivity of items	Paediatric Advanced Care Quality of Life Scale (PAC-QoL)	Not provided	Not applicable	Highlights importance of parent and healthcare provider input in QoL assessment tool development

The domain coverage matrix maps each tool to key outcome areas, revealing strong representation for symptom burden and psychological well-being, but limited coverage of spiritual health, caregiver burden and end-of-life care. It highlights critical gaps in holistic, cancer-specific assessment tools for paediatric palliative care.

### Outcome domains and tools identified

A wide array of outcome measurement tools was identified and grouped according to the primary outcome domains they assessed:

#### Symptom burden and distress

Symptom-related outcomes were the most frequently assessed. Tools such as the Paediatric Quality of Life & Evaluation of Symptoms—Memorial Symptom Assessment Scale (PediQUEST-MSAS) [12, 31, 35–37], Symptom Screening in Pediatrics Tool (SSPedi) [30, 39] and Memorial Symptom Assessment Scale (MSAS 10–18) [29, 43] were commonly used to evaluate physical and psychological symptoms. The SSPedi demonstrated strong psychometric reliability, with a Cronbach’s alpha of 0.87 and high inter-rater reliability [39]. Other tools, like the Perceived Symptom Severity (PSS) and Edmonton Symptom Assessment Scale (ESAS), were also used [49, 51] though evidence for paediatric-specific validation was limited.

#### Quality of Life (QoL)

Multiple QoL tools were used, including both generic and cancer-specific modules. The Paediatric Quality of Life Inventory (PedsQL 4.0 [2, 27, 31, 36] and 3.0 Cancer Module [28, 30, 32, 43]) and the newly developed Advance QoL PROM were prominently featured [41]. The Paediatric Advanced Care Quality of Life Scale (PAC-QoL) was a comprehensive tool incorporating parent and child perspectives and demonstrated high internal consistency (*α* = 0.91) [38]. The Quality of Children’s Palliative Care Instrument (QCPCI) assessed parental perceptions of care quality with strong psychometric properties (*α* = 0.78–0.90) [42].

#### Psychological outcomes and coping

Psychological symptoms such as anxiety, depression, stress and coping mechanisms were assessed using tools like the Beck Anxiety Inventory (BAI) [2], Beck Depression Inventory II (BDI-II) [2, 34], Perceived Stress Scale (PSS) [34], Impact of Events Scale—Revised [32] and the Paediatric Cancer Coping Scale (PCCS) [51]. These tools demonstrated strong internal consistency; however, most were originally developed for adult populations and have only been partially validated in PPC contexts.

#### Spiritual and existential well-being

Tools such as the Functional Assessment of Chronic Illness Therapy—Spiritual Well-Being Scale (FACIT-Sp) [2] and the Spiritual Involvement and Beliefs Scale (SIBS) [34] were employed to evaluate aspects of meaning, peace and faith. Spirituality emerged as a key coping resource, particularly among adolescents and their caregivers, contributing positively to emotional well-being and resilience in the PPC setting.

#### Caregiver burden and communication

Caregiver experiences were assessed using tools such as the Inventory of Traumatic Grief [47], Ruminative Response Scale [32] and the Survey about Caring for Children with Cancer (SCCC) [33, 40, 48]. Studies highlighted the significant psychological toll on parents, with grief, stress and persistent rumination commonly reported. Importantly, supportive and effective communication was shown to play a protective role, with improved communication linked to reduced long-term grief and more favourable perceptions of end-of-life care [47].

#### End-of-Life (EOL) outcomes

EOL care quality was evaluated using tools such as the Good Death Inventory—Paediatrics (GDI-P) and the Needs at the End-of-Life Screening Tool (NEST) [43, 44]. These tools captured key dimensions of end-of-life care, including preparedness for death, symptom management, spiritual well-being and relational factors. Several were adapted for proxy reporting by nurses and parents, offering valuable perspectives on the child’s EOL experience and family satisfaction with care delivery.

A summary matrix comparing psychometric strengths and limitations showed that tools for symptom burden and QoL (e.g. SSPedi, PedsQL) were well-validated in paediatric populations. Psychological tools had strong reliability but were mostly adapted from adult versions. Spiritual and caregiver burden tools showed good internal consistency but lacked cultural adaptation for LMICs. End-of-life tools (e.g. GDI-P, NEST) were valid but relied on proxy reporting. Overall, while some domains are well-covered, gaps remain in culturally adapted, cancer-specific tools for PPC (Table 4).

**Table 4:** Study design and methodology.

Study information		Methodology		
S.no	Authors & year	Study design	Data collection tools	Follow-up period
1	Murti Andriastuti et al. 2024	Likely cross-sectional/observational	the PedsQL for quality-of-life assessment, FACES III for evaluating family functioning and the SSERQ for measuring family cancer-related emotions	Not mentioned
2	Cowfer et al. 2024	Cross-sectional study	Surveys and questionnaires administered during clinic visits	None stated
3	Cadamuro et al. 2024	Descriptive, cross-sectional study	Interviews, medical records, PedsQL and SSPedi-BR questionnaires	None stated
4	Grinde et al. 2024	Longitudinal, prospective, descriptive	Paediatric Patient-Reported Outcome Common Terminology Criteria for Adverse Events (Ped PRO-CTCAE®) & PedsQL™ Cancer Module 3.0	6 months
5	Hamner et al. 2024	Prospective, observational	PedsQL (emotional, physical, social, and school functioning domains); parental chronic stress assessments	N/A
6	Wolfe et al. 2024	Prospective cohort study (embedded in PediQUEST RCT)	PQ-MSAS symptom assessment, collected via electronic PRO system (self and proxy reports); clinical data obtained from medical records	9 months
7	M Sarah et al. 2024	Longitudinal qualitative interview study	In-depth interviews	13 months, three interviews per family
8	Sharp et al. 2023	Cross-sectional study	Surveys completed during hospital visits and individual appointments	N/A
9	Zhong et al. 2023	Cross-sectional survey	PPCAS questionnaire, Qualtrics & WJX platforms	No follow-up period mentioned
10	Joana Duran, DrNP, et al. 2021	Longitudinal, descriptive, exploratory.	Paediatric Quality of Life Inventory (PedsQL™), Adolescent Paediatric Pain Tool (APPT).	Third day post-discharge
11	Hendriks et al. 2021	Qualitative study with semistructured interviews	Audio-recorded interviews	Time since end of treatment: ≥2 years
12	Ghoshal A et al. 2021	Retrospective 5-year audit.	Review of medical case records (inpatient and outpatient), as well as analysis of electronic medical records.	5 years
13	Erika Juškauskienė, RN et al. 2021	Cross-sectional quantitative study design.	Structured surveys administered by paediatric nurses; face-to-face interviews.	Not specified
14	Erica C. Kaye et al. 2021	Retrospective cohort study.	Standardised data extraction tool (67,308 cells) used to gather comprehensive clinical data; data from electronic medical records.	Not specified
15	Mitchell S, 2021	Longitudinal qualitative study	In-depth, semi-structured interviews with children and families	13 months
16	Schneider A, Wurz A, Vogel A, 2021	Cross-sectional study	Questionnaires, puppet interviews for younger children	Not specified
17	Snaman J et al. 2020	Review or conceptual framework likely; specific design not detailed.	Review of existing literature, case studies and clinical practices; interviews may have been included	Not specified
18	Sung L, Zaoutis T, Ullrich CK, 2019	Prospective, multicenter study as part of AAML1031 trial	PedsQL questionnaires completed on paper in clinic/hospital settings	Baseline assessment within 14 days of starting Induction 1, followed by assessments post-Induction and Intensification cycles
19	Abby R. Rosenberg et al. 2016	Prospective cohort study	PediQUEST quality of life survey, child and parent reports	Follow-up varied based on individual patient prognosis, but generally focused on later stages of illness
20	Tamires Vieira Carneiro 2016	Study design not specified; likely a cross-sectional study or qualitative assessment.	not specified	not mentioned
21	Momani T.G., Mandrell B.N., Gattuso J.S., et al. 2016	Quantitative content analysis of qualitative data	Two interview questions: ‘What makes a good day for you?’ and ‘How has being sick been for you?’	6 treatment time points
22	Lubis et al. 2015	Cross-sectional study	PedsQL 3.0 & 4.0 questionnaires	None stated
23	Aeltsje Brinksma et al. 2014	Retrospective pre- and post-test design	Play Performance Scale, Memorial Symptom Assessment Scale, PedsQL, Cantril’s ladder	3 months post-diagnosis
24	Wolfe et al. 2014	Parallel, multicenter pilot randomised controlled trial	Computer-based PediQUEST survey	20 weeks
25	Banayat, Campos, et al. 2024	Embedded mixed-method design	Revised Cancer Patient Needs Assessment: Parent Perspective Tool (rCPNQ), Semi-structured interviews	No specific follow-up period mentioned
26	Nina Mogensen et al. 2024	Retrospective cohort study	PedsQL questionnaires, study-specific questionnaires; NOPHO ALL2008 database	≥6 months post-treatment
27	Nair et al. 2024	Retrospective study based on case records	Symptom tracking and referral times to palliative care	10 years
28	Pangarso et al. 2024	Cross-sectional study using semi-structured interviews	Cross-sectional study using semi-structured interviews	Interviews conducted between October 2019 and April 2020
29	Brian T. Cheng, Tenzin Wangmo 2024	Cross-sectional	National Inpatient Sample database (ICD-9-CM codes)	None
30	Kimberley Widger et al. 2024	Retrospective cohort	Paediatric Oncology Group of Ontario Networked Information System (POGONIS)	2000–2012
31	Silva, A.V.M.V. et al. 2023	Cross-sectional study	PedsQL™ 3.0, questionnaires on demographics and health	NA
32	Yang Chen et al. 2022	Retrospective cohort study	Electronic medical records	2012–2019
33	Trupti Narnaware, Maninderdeep Kaur, Sukhpal Kaur, Renu Madan 2022	Descriptive study	Sociodemographic & clinical profile sheets, Problem assessment proforma, PedsQL 4.0 Scale	July–September 2022
34	Russell et al. 2021	Cross-sectional descriptive, psychosocial assessment	PATrev (family risk), ESAS-r (symptom burden), CPC (concerns)	24 months
35	Song, I.G. et al. 2021	Retrospective cohort study	Patient hospital records, PaPaS evaluation	NA
36	Robson, P. C., Dietrich, M. S., Akard, T. F. 2021	Secondary analysis of RCT	Research Electronic Data Capture (REDCap)	Baseline assessment only
37	Cheryl Rodgers et al. 2021	Prospective, repeated-measures	Symptom questionnaires for fatigue, sleep disturbance, pain, nausea, depression; HRQOL questionnaire	Approximately 12 months
38	Jennifer L. Raybin et al. (2021)	Cross-sectional secondary analysis of prospective data	PedsQL, Faces Scale, inclinometer for posture	Baseline only
39	Nogueira A. et al. 2020	Retrospective cohort study design to analyze existing clinical records.	Clinical records, demographic and clinical data forms, questionnaires (if used) and standardised assessment tools for quality of life.	Length of time over which data was collected, or patients were monitored after initial assessment; in this case, it could be over the last year of life leading up to death.
40	Veronica Dussel et al. 2020	Cohort study embedded in an RCT	Medical records, survey tracking for pre-inclusion and post-inclusion attrition, and surveys to evaluate retention, reasons for participation and intermittent attrition (IA) patterns	9 months
41	Marieke Van Schoors et al. 2019	Longitudinal survey study	PSS, FES, SSERQ, MMQ	Diagnosis to 2.5 years post-diagnosis
42	Hamner, Latzman, Elkin, & Majumdar 2018	Observational study	Everyday Stressors Index (ESI), Paediatric Quality of Life Inventory (PedsQL)	Single assessment period (data collected from Winter 2009 to Fall 2010)
43	Olagunju et al. 2016	Cross-sectional study	Questionnaires, MSAS, CES-D-R	Not specified
44	Veronica Dussel, MD, MPH, et al. 2015	Cohort study embedded in a pilot RCT.	Consent Survey, Participation Survey, Paediatric Quality of Life Inventory (PedsQL™), patient/parent-reported outcomes using electronic systems (PediQUEST).	9 months (proposed)
45	Wolfe, J. 2015	Prospective cohort study embedded in a multisite randomised controlled trial	PQ-MSAS administered electronically	9 months with periodic assessments
46	Compas et al. 2014	Cross-sectional	Self-reports and parent reports of coping and distress	1.30 months post-diagnosis
47	Lee Ai Chong et al. 2014	Observational cross-sectional study	Medical case notes, anonymized data entry	2014
48	Joanne Wolfe, et al. 2014	Randomised Controlled Trial (RCT), pilot, multicenter	PediQUEST electronic surveys: PedsQL4.0, PQ-MSAS, Sickness Question, satisfaction surveys for children, parents and providers	Up to 3 months, with multiple reenrollments allowed
49	Danielle Cataudella, PsyD, et al. 2014	Instrument development study, content validation	Construct and item generation, expert reviews	Not applicable

## Discussion

This scoping review highlights several critical issues in the current landscape of outcome measurement tools in PPC for patients with cancer. While there is a commendable focus on symptom burden and quality of life, other equally important domains, such as psychological well-being, spiritual health, caregiver burden and end-of-life preparedness, are often underrepresented [52, 53]. Tools like SSPedi [30] and PedsQL^TM53^ have demonstrated robust psychometric properties and clinical utility; however, their usage remains predominantly confined to high-income countries and structured hospital environments.

The heavy reliance on adult-derived tools or those lacking paediatric-specific validation is a major limitation. Psychological and spiritual assessments often utilise measures that were originally designed for adults or general paediatric populations, without adequate adaptation for the unique needs of children with advanced cancer. Such tools may fail to capture the nuanced experiences of these children, including anticipatory grief, existential concerns, or context-specific stressors. This shortfall is especially concerning in LMICs, where spiritual and cultural dimensions are often central to the caregiving experience [52–54].

Another significant finding is the persistent use of proxy reporting. While necessary in cases involving very young or critically ill children, exclusive dependence on caregiver or clinician proxies may obscure the child’s lived experiences. Children’s voices, especially in the domains of pain, emotional state and preferences, must be foregrounded wherever possible. Encouragingly, digital innovations and simplified PRO platforms now offer age-appropriate methods for symptom self-reporting, even in young populations [53–55].

The contextual gap is also striking. Of the 28 tools identified, only four were adapted or validated in LMICs. This raises serious concerns about cultural and linguistic relevance, particularly in regions where healthcare delivery models, social norms and family dynamics differ significantly from Western contexts. The study by Namisango et al. [56] provides a rare example of participatory tool development in LMICs, emphasising the importance of co-designing tools with local stakeholders. Such participatory methodologies enhance not only the acceptability of tools but also their sensitivity to the sociocultural and ethical realities of care [57].

Additionally, caregiver and family-centred outcome measures remain sparse. Caregivers in paediatric oncology bear immense psychological and emotional burdens. Their perspectives on care quality, communication, decision-making and spiritual needs are vital for a comprehensive evaluation of palliative care outcomes. Integrating caregiver-reported outcomes alongside child self-report measures can create a fuller picture of care experiences and inform family-centred care strategies.

Ultimately, the review underscores the need for multidimensional tools that accurately capture the complexity of the paediatric palliative experience. We categorised each tool based on its primary intended construct as defined by the original developers, acknowledging that many tools (e.g. PedsQL Cancer Module) include overlapping domains such as symptoms and psychological distress. Future research should focus on developing integrated instruments that assess physical, emotional, spiritual and familial dimensions in a cohesive manner. Interdisciplinary collaboration among clinicians, psychologists, social workers, and communities will be vital to ensure the development of tools that are not only psychometrically sound but also practical and humane.

### Limitations

This review has several limitations, including that most studies were conducted in high-income countries and that few tools were validated in LMICs or community settings. Additionally, several tools lacked specificity for paediatric populations, and the reporting of psychometric properties was inconsistent. There was also no formal quality appraisal conducted, following the methodology for scoping reviews. Furthermore, our English-only search and exclusion of grey literature may have disproportionately limited the identification of LMIC tools, as outcome measure development is often documented in reports, theses, or locally published journals.

## Conclusion

This review highlights the urgent need for validated contextually relevant outcome measurement tools in paediatric palliative oncology. We situate our findings within the broader literature, which shows an increasing interest in PRO implementation in LMICs, aligned with emerging frameworks for culturally grounded paediatric PRO development. While existing tools have made progress in assessing symptom burden and quality of life, several critical areas remain underrepresented. These include psychological resilience, spiritual well-being, caregiver distress and cultural values. It is essential to develop tools that are inclusive, adaptable and participatory. Such tools should effectively capture the unique experiences of children and families dealing with life-limiting illnesses, especially in LMICs where resources are scarce and the caregiving burden is significant. Co-creation with end users—children, caregivers and local clinicians—will help ensure that these tools are ethically grounded, culturally appropriate and practical to implement.

Additionally, it is crucial to integrate these tools into routine clinical practice. Health systems should be prepared to adopt validated tools for real-time decision-making, quality improvement and research purposes. Investments in training, digital infrastructure and interdisciplinary care models will support meaningful implementation. Ultimately, establishing a global consensus framework for paediatric palliative outcome measurement—focused on equity, child voice and cultural sensitivity—will be vital for advancing palliative care innovation. The way forward involves combining scientific rigor with compassionate care, ensuring that every child with cancer receives support that is measurable, meaningful and responsive to their needs.

## Supplementary information

supplementary file 1

supplementary file II

## Data Availability

All data supporting the findings of this study are available within the paper.
