# Supplementary material for: Outcome measurement tools in pediatric oncology palliative care: a scoping review of domains, validation and contextual relevance
Source: BJC Rep. 2026 Jun 16;4:33. doi: 10.1038/s44276-026-00219-9 (PMC13273101; doi:10.1038/s44276-026-00219-9)
Supplement: Supplementary file 1 [file 44276_2026_219_MOESM1_ESM.docx]

**Supplementary file I**

**PRISMA-ScR Checklist (Completed)**

**Title of review:** *Outcome Measurement Tools in Pediatric Oncology Palliative Care: A Scoping Review of Domains, Validation, and Contextual Relevance*
**Type:** Scoping Review
**Registration:** OSF (<https://doi.org/10.17605/OSF.IO/G8BN3>)

**SECTION 1: TITLE**

| **PRISMA-ScR Item** | **Description** | **Reported Where** |
| --- | --- | --- |
| **1. Title** | Identify the report as a scoping review. | Title page, Abstract |

**SECTION 2: ABSTRACT**

| **Item** | **Description** | **Reported Where** |
| --- | --- | --- |
| **2. Structured Summary** | Structured summary including background, objectives, eligibility criteria, data sources, charting methods, results, and conclusions. | Abstract |

**SECTION 3: INTRODUCTION**

| **Item** | **Description** | **Reported Where** |
| --- | --- | --- |
| **3. Rationale** | Describe rationale for the review in context of existing knowledge. | Introduction (pages 1–3) |
| **4. Objectives** | State the objectives or questions the scoping review addresses. | Introduction, Stage 1 (page 4) |

**SECTION 4: METHODS**

| **Item** | **Description** | **Reported Where** |
| --- | --- | --- |
| **5. Protocol & Registration** | Indicate whether a protocol exists. | Methods – PRISMA-ScR Statement & OSF link (page 4) |
| **6. Eligibility Criteria** | Specify characteristics of sources of evidence used as eligibility criteria. | Methods – Stage 2 (pages 5–6) |
| **7. Information Sources** | Describe all information sources and date of search. | Methods – Stage 2 (page 5) |
| **8. Search** | Present full electronic search strategy. | Supplementary File II (referenced in Methods) |
| **9. Selection of Sources** | State the process for selection (screening, eligibility, inclusion). | Methods – Stage 3 (page 6) |
| **10. Data Charting Process** | Describe methods of data extraction/charting. | Methods – Stage 4 (page 7) |
| **11. Data Items** | List all variables/data categories sought. | Methods – Stage 4 (page 7) |
| **12. Critical Appraisal (if done)** | Not required for scoping reviews; not conducted. | Not applicable (not performed) |
| **13. Synthesis of Results** | Describe methods of handling, summarizing, and reporting results. | Methods – Stage 5 (page 8) |

**SECTION 5: RESULTS**

| **Item** | **Description** | **Reported Where** |
| --- | --- | --- |
| **14. Selection of Sources** | Provide numbers of included/excluded records with reasons. | Results – Study Characteristics, PRISMA flow narrative (page 9) |
| **15. Characteristics of Included Sources** | Present characteristics for each source. | Results – Table 1, Table 2 (pages 10–30) |
| **16. Critical Appraisal Within Sources** | If done—NOT APPLICABLE for your review. | Not applicable |
| **17. Results of Individual Sources** | Present data relevant to research questions. | Results, Tables 1–3 |
| **18. Synthesis of Results** | Summarize charted results in relation to review questions. | Results – narrative synthesis (pages 9–30) |

**SECTION 6: DISCUSSION**

| **Item** | **Description** | **Reported Where** |
| --- | --- | --- |
| **19. Summary of Evidence** | Summarize main results, including concepts, implications, and gaps. | Discussion |
| **20. Limitations** | Discuss limitations of the scoping review process. | Discussion (final paragraph) |
| **21. Conclusions** | Provide interpretation and implications for research/practice. | Conclusions |

**SECTION 7: FUNDING**

| **Item** | **Description** | **Reported Where** |
| --- | --- | --- |
| **22. Funding** | Describe funding sources for review and included studies. | Manuscript: Funding statement (if not included, add during revision) |
