# Supplementary material for: Outcome measurement tools in pediatric oncology palliative care: a scoping review of domains, validation and contextual relevance
Source: BJC Rep. 2026 Jun 16;4:33. doi: 10.1038/s44276-026-00219-9 (PMC13273101; doi:10.1038/s44276-026-00219-9)
Supplement: Supplementary file 2 — supplementary file II [file 44276_2026_219_MOESM2_ESM.pdf]

Supplementary Table S1: Search Strategy

Search Strategy include PubMed (MEDLINE), EMBASE, CINAHL (EBSCOhost), Scopus (Elsevier), Web of Science (Clarivate), ProQuest, and grey literature databases

PubMed (MEDLINE)

S. No	Query	Filters applied	No. of hits	Date of search
#1	("Neoplasms"[MeSH Terms] OR "neoplasm*"[Title/Abstract] OR "cancer*"[Title/Abstract] OR "leukemia*"[Title/Abstract] OR "leukaemia*"[Title/Abstract] OR "malignan*"[Title/Abstract] OR "tumor*"[Title/Abstract] OR "tumour*"[Title/Abstract] OR "Childhood Cancer"[Title/Abstract] OR "Pediatric Cancer"[Title/Abstract])		5314951	22.08.2024
#2	"Child"[MeSH Terms] OR "child, preschool"[MeSH Terms] OR "Pediatrics"[MeSH Terms] OR "Infant"[MeSH Terms] OR "Adolescent"[MeSH Terms] OR "child*"[Title/Abstract] OR "infant*"[Title/Abstract] OR "teen*"[Title/Abstract] OR "youth"[Title/Abstract] AND "adolescen*"[Title/Abstract]		355894	
#3	"Palliative Care"[MeSH Terms] OR "Quality of Life"[MeSH Terms] OR "Symptom Management"[Title/Abstract] OR "Family"[MeSH Terms] OR "Needs Assessment"[MeSH Terms] OR "Quality of Life"[Title/Abstract] OR "symptom relief"[Title/Abstract] OR "supportive care"[Title/Abstract]		949220	
#4	#1 AND #2 AND #3	Language: English Year:	2214	

		2000-2024 Full textSpecies: Humans		

EMBASE

S. No	Query	No. of Hits	Date of search
#1	'neoplasm'/exp OR 'neoplasm' OR 'neoplasm*':ti,ab OR 'cancer*':ti,ab OR 'leukemia*':ti,ab OR 'leukaemia*':ti,ab OR 'malignan*':ti,ab OR 'tumor*':ti,ab OR 'tumour*':ti,ab OR 'childhood cancer':ti,ab OR 'pediatric cancer':ti,ab	7500371	22.08.2024
#2	('child'/exp OR 'preschool child'/exp OR 'pediatrics'/exp OR 'infant'/exp OR 'adolescent'/exp OR 'child*':ti,ab OR 'infant*':ti,ab OR 'teen*':ti,ab OR 'youth':ti,ab) AND 'adolescen*':ti,ab	453505	
#3	'palliative therapy'/exp OR 'quality of life'/exp OR 'symptom management':ti,ab OR 'family'/exp OR 'needs assessment'/exp OR 'quality of life':ti,ab OR 'symptom relief':ti,ab OR 'supportive care':ti,ab	1659617	
#4	#1 AND #2 AND #3	6277	
#5	#1 AND #2 AND #3 AND [english]/lim AND [2014-2024]/py	4078	

CINAHL (EBSCOhost)

S. No	Query	Filters applied	No. of hits	Date of search
S1	((MH Neoplasms+) OR (TI neoplasm* OR AB neoplasm*) OR (TI cancer* OR AB cancer*) OR (TI leukemia* OR AB		9,308,438	22.08.2024

	leukemia*) OR (TI leukaemia* OR AB leukaemia*) OR (TI malignan* OR AB malignan*) OR (TI tumor* OR AB tumor*) OR (TI tumour* OR AB tumour*) OR (TI "Childhood Cancer" OR AB "Childhood Cancer") OR (TI "Pediatric Cancer" OR AB "Pediatric Cancer"))			
S3	(MH Child+) OR (MH "child, preschool+") OR (MH Pediatrics+) OR (MH Infant+) OR (MH Adolescent+) OR (TI child* OR AB child*) OR (TI infant* OR AB infant*) OR (TI teen* OR AB teen*) OR (TI youth OR AB youth) AND (TI adolescen* OR AB adolescen*)		6,663,390	
S4	(MH "Palliative Care+") OR (MH "Quality of Life+") OR (TI "Symptom Management" OR AB "Symptom Management") OR (MH Family+) OR (MH "Needs Assessment+") OR (TI "Quality of Life" OR AB "Quality of Life") OR (TI "symptom relief" OR AB "symptom relief") OR (TI "supportive care" OR AB "supportive care")		1,632,601	
S5	S1 AND S2 AND S3	Limiters - Publication Date: 20000101-20241231 Expanders - Apply equivalent subjects Narrow by Language: - english Search modes - Proximity	1350	

Scopus (Elsevier)

S. No	Query	No. of hits	Date of search
#1	(INDEXTERMS(Neoplasms) OR TITLE-ABS(neoplasm*) OR TITLE-ABS(cancer*) OR TITLE-ABS(leukemia*) OR TITLE-ABS(leukaemia*) OR TITLE-ABS(malignan*) OR TITLE-ABS(tumor*) OR TITLE-ABS(tumour*) OR TITLE-ABS("Childhood Cancer") OR TITLE-ABS("Pediatric Cancer"))	5,626,938	22.08.2024
#2	INDEXTERMS (child) OR INDEXTERMS ("child, preschool") OR INDEXTERMS (pediatrics) OR INDEXTERMS (infant) OR INDEXTERMS (adolescent) OR TITLE-ABS (child*) OR TITLE-ABS (infant*) OR TITLE-ABS (teen*) OR TITLE-ABS (youth) AND TITLE-ABS (adolescen*)	439,231	
#3	INDEXTERMS ("Palliative Care") OR INDEXTERMS ("Quality of Life") OR TITLE-ABS ("Symptom Management") OR INDEXTERMS (family) OR INDEXTERMS ("Needs Assessment") OR TITLE-ABS ("Quality of Life") OR TITLE-ABS ("symptom relief") OR TITLE-ABS ("supportive care")	1,482,539	
#4	#1 AND #2 AND #3	3,551	
#5	(INDEXTERMS(Neoplasms) OR TITLE-ABS(neoplasm*) OR TITLE-	1,523	

	ABS(cancer*) OR TITLE-ABS(leukemia*) OR TITLE-ABS(leukaemia*) OR TITLE-ABS(malignan*) OR TITLE-ABS(tumor*) OR TITLE-ABS(tumour*) OR TITLE-ABS("Childhood Cancer") OR TITLE-ABS("Pediatric Cancer")) AND INDEXTERMS (child) OR INDEXTERMS ("child, preschool") OR INDEXTERMS (pediatrics) OR INDEXTERMS (infant) OR INDEXTERMS (adolescent) OR TITLE-ABS (child*) OR TITLE-ABS (infant*) OR TITLE-ABS (teen*) OR TITLE-ABS (youth) AND TITLE-ABS (adolescen*) AND INDEXTERMS ("Palliative Care") OR INDEXTERMS ("Quality of Life") OR TITLE-ABS ("Symptom Management") OR INDEXTERMS (family) OR INDEXTERMS ("Needs Assessment") OR TITLE-ABS ("Quality of Life") OR TITLE-ABS ("symptom relief") OR TITLE-ABS ("supportive care") AND PUBYEAR > 2013 AND PUBYEAR < 2025 AND (LIMIT-TO (DOCTYPE,"ar")) AND (LIMIT-TO (EXACTKEYWORD,"Human") OR LIMIT-TO (EXACTKEYWORD,"Article")) AND (LIMIT-TO (LANGUAGE,"English"))		

<u>Web of Science (Clarivate)</u>

S. No	Query	No. of hits	Date of search
#1	ALL=((Neoplasms OR neoplasm* OR cancer* OR leukemia* OR leukaemia* OR malignan* OR tumor* OR tumour* OR "Childhood Cancer" OR "Pediatric Cancer"))	5,436,287	22.08.2024
#2	ALL=(Child OR "child, preschool" OR Pediatrics OR Infant OR Adolescent OR child* OR infant* OR teen* OR youth AND adolescen*)	4,018,601	
#3	ALL=("Palliative Care" OR "Quality of Life" OR "Symptom Management" OR Family OR "Needs Assessment" OR "Quality of Life" OR "symptom relief" OR "supportive care")	3,012,926	
#4	#3 AND #2 AND #1	58,395	
#5	#3 AND #2 AND #1 and 2014 or 2015 or 2016 or 2017 or 2018 or 2019 or 2020 or 2021 or 2022 or 2023 or 2024 (Final Publication Year) and Article (Document Types) and Open Access and English (Languages)	19,596	

ProQuest

S. No	Query	Filters applied	No. of hits	Date of search
S1	("Neoplasms"[MeSH Terms] OR "neoplasm*"[Title/Abstract] OR "cancer*"[Title/Abstract] OR "leukemia*"[Title/Abstract] OR "leukaemia*"[Title/Abstract] OR "malignan*"[Title/Abstract] OR "tumor*"[Title/Abstract] OR "tumour*"[Title/Abstract] OR "Childhood Cancer"[Title/Abstract] OR "Pediatric Cancer"[Title/Abstract])		15,557	22.08.2024
S2	"Child"[MeSH Terms] OR "child, preschool"[MeSH Terms] OR "Pediatrics"[MeSH Terms] OR "Infant"[MeSH Terms] OR "Adolescent"[MeSH Terms] OR "child*"[Title/Abstract] OR		38,131	

	"infant*"[Title/Abstract] OR "teen*"[Title/Abstract] OR "youth"[Title/Abstract] AND "adolescen*"[Title/Abstract]			
S3	"Palliative Care"[MeSH Terms] OR "Quality of Life"[MeSH Terms] OR "Symptom Management"[Title/Abstract] OR "Family"[MeSH Terms] OR "Needs Assessment"[MeSH Terms] OR "Quality of Life"[Title/Abstract] OR "symptom relief"[Title/Abstract] OR "supportive care"[Title/Abstract]		77,349	
S4	[S1] AND [S2] AND [S3]	Language: English Year: 2014-2024	2,613	
